# Spatial-spectral fusion enables drug repositioning by capturing indirect and long-range associations in biological networks

**DOI:** 10.1093/bioinformatics/btag553

**Published:** 2026-08-10

**Authors:** Xiaobo Zhu, Lei Wang, Runzhou Tang, Zhi-An Huang, Yu-an Huang, Feng Tan, Lun Hu, Zhuhong You, Pengwei Hu

**Affiliations:** Xinjiang Technical Institute of Physics and Chemistry, Chinese Academy of Sciences, Urumqi 810011, China; China University of Mining and Technology, Xuzhou, China; Xinjiang Technical Institute of Physics and Chemistry, Chinese Academy of Sciences, Urumqi 810011, China; Research Office, City University of Hong Kong (Dongguan), Dongguan 523000, China; School of Computing, Northwestern Polytechnical University, Xi’an 710129, China; AI and Quantum Lab, Darmstadt, Germany; Xinjiang Technical Institute of Physics and Chemistry, Chinese Academy of Sciences, Urumqi 810011, China; School of Computing, Northwestern Polytechnical University, Xi’an 710129, China; Xinjiang Technical Institute of Physics and Chemistry, Chinese Academy of Sciences, Urumqi 810011, China

## Abstract

**Motivation:**

Drug repositioning accelerates clinical translation by identifying new therapeutic indications for approved drugs. However, therapeutic associations in biomolecular networks often exist indirectly, through transitive chains and long-range mechanisms, rather than as directly observed links. Shallow methods are confined to direct similarity and miss such indirect associations, whereas deep graph neural networks suffer from over-smoothing and lose discriminative power in highly connected networks.

**Results:**

We propose a spatial-spectral collaborative framework. In the spatial domain, a wave-evolution process propagates similarity from local to global, capturing multi-hop transitive associations while preserving discriminative representations. In the spectral domain, network-specific spectral transforms model global connectivity for long-range dependencies over homogeneous similarity and heterogeneous drug-protein-disease networks, with the two views aligned by contrastive learning. On three benchmarks the method outperforms state-of-the-art baselines on most evaluation metrics; case studies on Alzheimer’s and Parkinson’s disease and molecular docking confirm its ability to recover non-explicit therapeutic associations.

**Availability:**

The source code and data are available at https://github.com/Juniper-cola/BIO_SSF.

## Graphical Abstract

**Figure:**
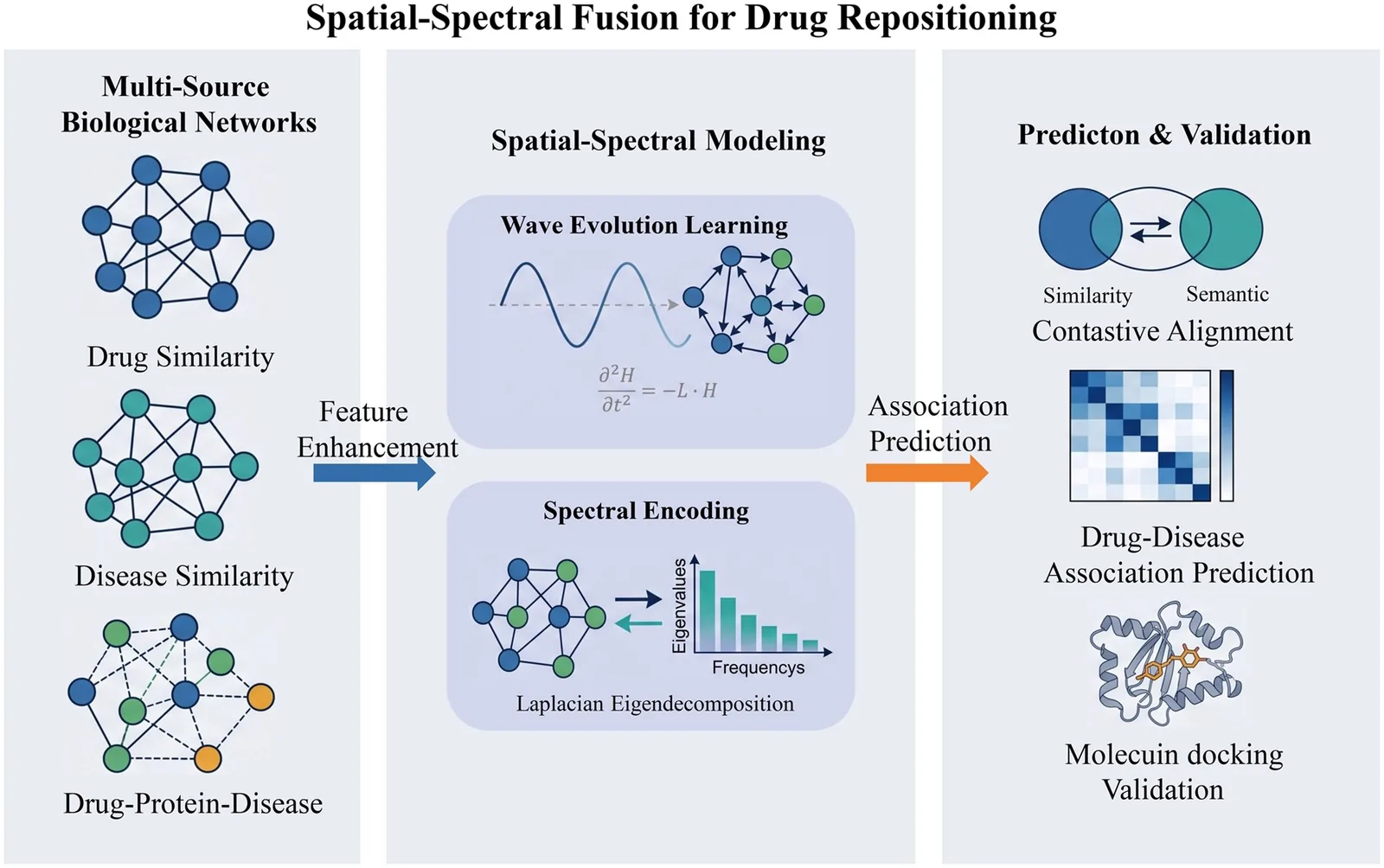

## 1 Introduction

Drug repositioning reduces development cycles and clinical risks by predicting new therapeutic indications for approved drugs (Huang *et al*. 2024a, Zhang *et al*. 2025b). High-throughput biotechnology has generated massive biomedical data for computational approaches (Wong *et al*. 2023, He *et al*. 2024, Liao *et al*. 2025, Zheng *et al*. 2025). However, a core challenge remains overlooked: therapeutic associations often exist indirectly rather than being directly observable. In similarity networks, potential therapeutic opportunities arise through transitive relationships rather than direct similarity (Song *et al*. 2024). This pattern of indirect associations is pervasive in biological networks and represents a critical clue for discovering non-explicit therapeutic relationships. Moreover, drug mechanisms typically operate via complex long-range pathways. In drug-protein-disease heterogeneous networks, drugs target proteins (Zhang *et al*. 2025a, 2025c) that regulate signaling pathways and biological processes, ultimately affecting disease phenotypes. These mechanisms manifest as long-range paths spanning multiple node types, making identification of such mechanistic associations crucial for predicting new indications.

Existing methods face fundamental limitations in identifying indirect therapeutic associations. Shallow network-based methods such as DTINet (Luo *et al*. 2017) and DeepDR (Zeng *et al*. 2019) are confined to direct similarity relationships. Deep GNN-based methods, including LAGCN (Yu *et al*. 2021), HDGAT (Huang *et al*. 2024b), DRHGCN (Cai *et al*. 2021), DRWBNCF (Meng *et al*. 2022), and AMDGT (Liu *et al*. 2024a), expand receptive fields through multi-layer stacking, but cause representations to become homogeneous in highly connected networks (Fu *et al*. 2022, Sun *et al*. 2024), losing discriminative power (Cai *et al*. 2021, Liu *et al*. 2024a). This over-smoothing fundamentally undermines models when exploring indirect associations. Heterogeneous GNN-based methods such as HINGRL (Zhao *et al*. 2022), RLFDDA (Zhang *et al*. 2022), and SFRLDDA (Zhao *et al*. 2023) propagate information through local neighborhood aggregation, expanding receptive fields by only one hop per layer. Graph contrastive learning methods, including SADR (Jin *et al*. 2024), SGCD (Yu *et al*. 2025), DRGCL (Jia *et al*. 2025), MICLE (Cui *et al*. 2025), and DRMAHGC (Liu *et al*. 2024b), alleviate data sparsity through self-supervised signals but still inherit locality limitations. This layer-by-layer propagation paradigm fundamentally limits direct capture of distant drug-protein-disease action chains.

To overcome these limitations, we propose a spatial-spectral collaborative modeling framework for identifying complex drug-disease therapeutic associations. In the spatial domain, we model similarity propagation as a wave evolution process. The second-order wave equation enables oscillatory information propagation, capturing similarity information from local to global while maintaining discriminative representations. In the spectral domain, we leverage spectral decomposition to transform network signals to spectral space, directly modeling global association patterns between nodes at arbitrary distances. Low-frequency components encode global structure, enabling capture of long-range mechanisms. The framework constructs a dual-view representation space where the homogeneous similarity view captures therapeutic patterns through transitive associations and the heterogeneous semantic view extracts mechanisms through molecular action chains. A detailed review of related computational drug repositioning, spectral graph methods, and graph contrastive learning is provided in Supplementary Appendix A, available as supplementary data at *Bioinformatics* online. The main contributions are summarized as follows:

- We propose a wave equation-based method that captures transitive associations from local to global through oscillatory propagation, effectively identifying indirect drug-disease associations while avoiding over-smoothing.
- We develop a spectral transformation method that directly models associations between distant nodes in both homogeneous and heterogeneous networks, enabling effective capture of long-range mechanisms without locality constraints.
- The proposed spatial-spectral framework integrates wave-based propagation with spectral modeling, unifying multi-scale network information from local to global for drug repositioning.

## 2 Methodology

**Figure 1:**
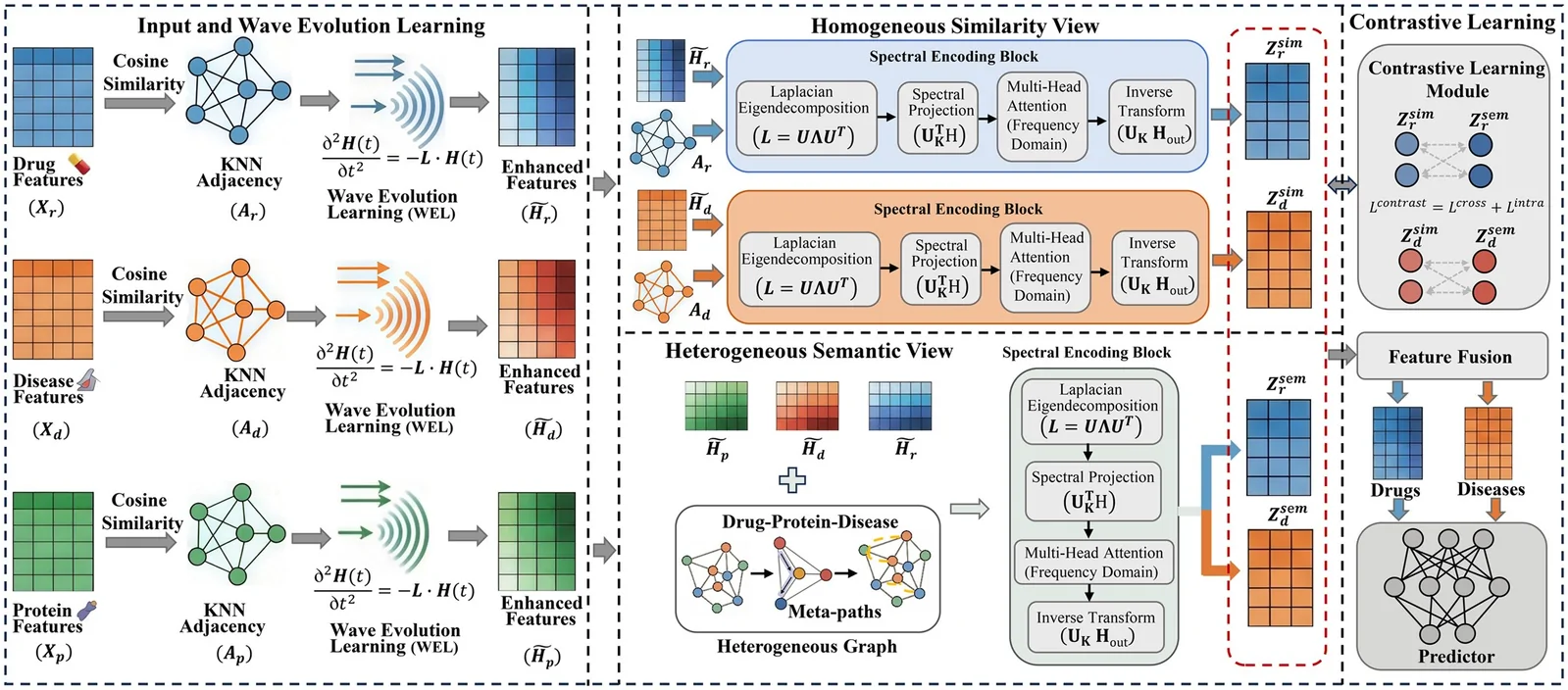
Overall architecture of the proposed model. Wave evolution learning captures multi-scale similarity patterns, dual-view spectral encoding models both homogeneous similarity networks and heterogeneous Drug-Protein-Disease networks, and contrastive learning aligns representations from both views. Finally, the fused representations are fed into a predictor to output association probabilities.

The proposed spatial-spectral collaborative modeling framework integrates similarity network construction with wave evolution-based feature enhancement, dual-view spectral graph encoding, and contrastive learning for association prediction (Fig. 1). Homogeneous similarity networks are constructed from molecular features, with wave evolution applied for multi-scale similarity modeling to capture transitive association signals. Dual spectral views are then constructed where the homogeneous view models drug-drug and disease-disease networks while the heterogeneous view encodes drug-protein-disease pathways. Contrastive learning integrates both views, fusing structural similarity and biological mechanism reasoning for unified predictions.

### 2.1 Wave evolution learning

Wave evolution is employed to model feature propagation in drug-drug and disease-disease similarity networks, capturing multi-scale transitive associations while maintaining discriminative node representations. For entities *i* and *j*, pairwise similarity is computed using cosine similarity:


(1) $$S(i,j)=\frac{X(i,:)\cdot X{\left(j,:\right)}^{T}}{\left\|X(i,:)\|\|X(j,:)\right\|}$$

Where *X* denotes the entity feature matrix. Similarity matrices $S_{r}\in R^{N_{r}\times N_{r}}$, $S_{d}\in R^{N_{d}\times N_{d}}$, and $S_{p}\in R^{N_{p}\times N_{p}}$ are computed for drugs, diseases, and proteins respectively. Sparse topological structures are constructed through a $K_{nn}$ nearest neighbor strategy that retains only the $K_{nn}$ most similar neighbors for each node: A(i,j)=1 when $j\in {\text{Top}}_{K_{nn}}(S(i,:))$ or i=j, otherwise A(i,j)=0.

The wave evolution equation for similarity propagation is defined as (Yue *et al*. 2025):


(2) $$\frac{\partial^{2}H(t)}{\partial t^{2}}=-LH(t)$$

Where H(t) represents node features at time *t*, and $L=I-D^{-1/2}AD^{-1/2}$ is the normalized graph Laplacian. Multi-scale similarity aggregation is obtained through recursive computation by discretizing the wave equation:


(3) $$H(t+\Delta t)=2H(t)-H(t-\Delta t)-\Delta t^{2}LH(t)$$

The initial conditions are set as $H(0)=XW_{0}$ and $H(-\Delta t)=H(0)-\Delta t\cdot XW_{1}$ where $W_{0},W_{1}$ are learnable transformations. Setting total evolution time as *T* and time step as Δt, the iteration count is n=⌈T/Δt⌉.

Original features are first projected to a unified hidden dimension: $H_{r}^{\left(0\right)}=\text{ELU}(X_{r}W_{r}^{\mathrm{proj}})$, $H_{d}^{\left(0\right)}=\text{ELU}(X_{d}W_{d}^{\mathrm{proj}})$, $H_{p}^{\left(0\right)}=\text{ELU}(X_{p}W_{p}^{\mathrm{proj}})$ where $W_{r}^{\mathrm{proj}}\in R^{d_{r}\times d_{h}}$, $W_{d}^{\mathrm{proj}}\in R^{d_{d}\times d_{h}}$, $W_{p}^{\mathrm{proj}}\in R^{d_{p}\times d_{h}}$ are projection matrices. Wave evolution is then applied: ${\tilde{H}}_{r}=\text{WEL}(H_{r}^{\left(0\right)},A_{r})$, ${\tilde{H}}_{d}=\text{WEL}(H_{d}^{\left(0\right)},A_{d})$, ${\tilde{H}}_{p}=\text{WEL}(H_{p}^{\left(0\right)},A_{p})$, yielding enhanced representations integrating multi-scale similarity information.

### 2.2 Spectral domain modeling

Spectral encoding transforms network signals from spatial to spectral domain through graph spectral decomposition, enabling direct modeling of associations between nodes at arbitrary distances. Customized modules are designed for homogeneous similarity networks and heterogeneous biological networks.

Given the normalized graph Laplacian $L=I-D^{-1/2}AD^{-1/2}$, spectral decomposition yields a Fourier basis $U=[u_{1},\ldots ,u_{N}]$. The first *K* eigenvectors are retained as spectral basis $U_{K}=[u_{1},\ldots ,u_{K}]$ where $K\in [K_{\min},K_{\max}]$ is dynamically determined. Node features are projected into spectral space:


(4) $$H^{\mathrm{freq}}=U_{K}^{T}H$$

Where $H^{\mathrm{freq}}\in R^{K\times d}$ is the spectral representation. Multi-head attention is computed in spectral space for each head *h*:


(5) $$\begin{array}{c} Q_{h}^{\mathrm{freq}}=H^{\mathrm{freq}}W_{h}^{Q},\;K_{h}^{\mathrm{freq}}=H^{\mathrm{freq}}W_{h}^{K},\\ V_{h}^{\mathrm{freq}}=H^{\mathrm{freq}}W_{h}^{V} \end{array}$$

(6) $${\text{Attn}}_{h}^{\mathrm{freq}}=\text{softmax}\left(\frac{Q_{h}^{\mathrm{freq}}{\left(K_{h}^{\mathrm{freq}}\right)}^{T}}{\sqrt{d_{h}}}\right)V_{h}^{\mathrm{freq}}$$

Where $W_{h}^{Q},W_{h}^{K},W_{h}^{V}\in R^{d\times d_{h}}$ are learnable parameters, and $d_{h}=d/H$ denotes the dimensionality of each attention head, *d* is the hidden dimension, and *H* is the number of attention heads. Outputs from $N_{h}$ attention heads are concatenated and projected:


(7) $$H_{\mathrm{out}}^{\mathrm{freq}}=[{\text{Attn}}_{1}^{\mathrm{freq}}\|{\text{Attn}}_{2}^{\mathrm{freq}}\|\cdots \|{\text{Attn}}_{N_{h}}^{\mathrm{freq}}]W^{O}$$

Where $W^{O}\in R^{N_{h}d_{h}\times d}$. The spectral output is transformed back to spatial domain: $H^{\mathrm{spatial}}=U_{K}H_{\mathrm{out}}^{\mathrm{freq}}$. Spectral and original features are integrated through weighted fusion:


(8) $$H^{\mathrm{enhanced}}=(1-\beta )H+\beta H^{\mathrm{spatial}}$$

Where β∈[0,1] controls spectral enhancement. Residual connections, layer normalization, and feed-forward networks are applied: $H^{\prime }=\text{LayerNorm}(H+H^{\mathrm{enhanced}})$, $H_{\mathrm{out}}=\text{LayerNorm}(H^{\prime }+\text{FFN}(H^{\prime }))$.

Independent spectral encoding modules are applied to drug and disease similarity networks: $Z_{r}^{\mathrm{sim}}={\text{FreqEnco}}_{r}({\tilde{H}}_{r},A_{r})$, $Z_{d}^{\mathrm{sim}}={\text{FreqEnco}}_{d}({\tilde{H}}_{d},A_{d})$ where $Z_{r}^{\mathrm{sim}}\in R^{N_{r}\times d_{\mathrm{out}}}$ and $Z_{d}^{\mathrm{sim}}\in R^{N_{d}\times d_{\mathrm{out}}}$ are final representations in the homogeneous similarity view.

For the heterogeneous network $G=(V,E,T_{V},T_{E})$ where V=R∪D∪P, network connectivity is enhanced through a meta-path augmentation strategy based on graphon estimation (Zhou *et al*. 2023). Features of different node types are concatenated as $H^{\mathrm{heter}}=[{\tilde{H}}_{r};{\tilde{H}}_{d};{\tilde{H}}_{p}]\in R^{(N_{r}+N_{d}+N_{p})\times d_{h}}$, and the heterogeneous graph is mapped to homogenized representation $G^{\mathrm{homo}}$. The spectral encoding process is applied with *K* dynamically determined. Drug and disease representations in the heterogeneous semantic view are extracted as $Z_{r}^{\mathrm{sem}}=\text{FreqHenco}({\tilde{H}}_{r},{\tilde{H}}_{d},{\tilde{H}}_{p},G)[1:N_{r},:]$ and $Z_{d}^{\mathrm{sem}}=\text{FreqHenco}({\tilde{H}}_{r},{\tilde{H}}_{d},{\tilde{H}}_{p},G)[N_{r}+1:N_{r}+N_{d},:]$, capturing long-range semantic associations spanning multiple biomolecules.

### 2.3 Feature alignment learning

Representations across homogeneous similarity and heterogeneous semantic views are aligned through a contrastive learning framework, fusing similarity-based and mechanism-based reasoning. Positive sample pair matrices $P_{r}\in {\left\{0,1\right\}}^{N_{r}\times N_{r}}$ and $P_{d}\in {\left\{0,1\right\}}^{N_{d}\times N_{d}}$ are constructed based on meta-path reachability and feature similarity.

The cross-view contrastive loss aligns representations of the same entity across both views. For drugs:


(9) $$\begin{array}{cc} L_{r}^{\mathrm{cross}}=-\frac{1}{N_{r}}\sum_{i=1}^{N_{r}}\;\text{log}\; & \\ \frac{\sum_{j=1}^{N_{r}}P_{r}(i,j)\cdot \;\text{exp}\;\left(\text{sim}(Z_{r}^{\mathrm{sem}}(i),Z_{r}^{\mathrm{sim}}(j))/\tau^{\mathrm{cross}}\right)}{\sum_{j=1}^{N_{r}}\;\text{exp}\;\left(\text{sim}(Z_{r}^{\mathrm{sem}}(i),Z_{r}^{\mathrm{sim}}(j))/\tau^{\mathrm{cross}}\right)} & \end{array}$$

Where sim(·,·) is cosine similarity and $\tau^{\mathrm{cross}}$ is temperature. The disease cross-view loss $L_{d}^{\mathrm{cross}}$ follows the same form. Total cross-view loss is $L^{\mathrm{cross}}=\lambda^{\mathrm{cross}}L_{r}^{\mathrm{cross}}+(1-\lambda^{\mathrm{cross}})L_{d}^{\mathrm{cross}}$.

The intra-view contrastive loss enhances consistency within each view. For drugs:


(10) $$\begin{array}{cc} L_{r}^{\mathrm{intra}}=-\frac{1}{N_{r}}\sum_{i=1}^{N_{r}}\;\text{log}\; & \\ \frac{\sum_{j=1}^{N_{r}}P_{r}(i,j)\cdot \;\text{exp}\;\left(\text{sim}(Z_{r}^{\mathrm{sim}}(i),Z_{r}^{\mathrm{sim}}(j))/\tau^{\mathrm{intra}}\right)}{\sum_{j=1}^{N_{r}}\;\text{exp}\;\left(\text{sim}(Z_{r}^{\mathrm{sim}}(i),Z_{r}^{\mathrm{sim}}(j))/\tau^{\mathrm{intra}}\right)} & \end{array}$$

The disease intra-view loss $L_{d}^{\mathrm{intra}}$ follows the same form. Total intra-view loss is $L^{\mathrm{intra}}=\lambda^{\mathrm{intra}}L_{r}^{\mathrm{intra}}+(1-\lambda^{\mathrm{intra}})L_{d}^{\mathrm{intra}}$. The final contrastive learning loss is:


(11) $$L^{\mathrm{contrast}}=L^{\mathrm{cross}}+L^{\mathrm{intra}}$$

### 2.4 Drug-disease association prediction

Representations from semantic and similarity views are concatenated and passed through linear layers with ReLU activation to obtain fused representations $Z_{r}\in R^{N_{r}\times d_{\mathrm{out}}}$ and $Z_{d}\in R^{N_{d}\times d_{\mathrm{out}}}$. Positive samples are derived from known associations, while negative samples are generated through hard negative mining by selecting nodes within 2-hop distance. For each drug-disease pair $\left(r_{i},d_{j}\right)$, the interaction representation is computed as $h_{ij}=Z_{r}(i,:)⊙Z_{d}(j,:)$. The MLP outputs two logits $o_{ij}$, and $p_{ij}=\text{softmax}(o_{ij})$. The classification loss is:


(12) $$L^{\mathrm{cls}}=-\frac{1}{\left|S\right|}\sum_{(i,j)\in S}\;\text{log}\;p_{ij,y_{ij}},$$

Where *S* is the training sample set, $y_{ij}\in \{0,1\}$ is the ground-truth label, and $p_{ij,y_{ij}}$ is the predicted probability of the true class. The final training objective is:


(13) $$L=\alpha L^{\mathrm{contrast}}+(1-\alpha )L^{\mathrm{cls}}$$

Where α∈[0,1] balances both components. The simplified overall training procedure for the spatial-spectral framework is presented in Supplementary Appendix H, available as supplementary data at *Bioinformatics* online.

## 3 Experiments

### 3.1 Datasets and baseline methods

We evaluate the proposed method on three benchmark datasets with varying scale and sparsity (0.93%–11.45%), including B-dataset (Zhang *et al*. 2018), C-dataset (Luo *et al*. 2016), and F-dataset (Gottlieb *et al*. 2011), and compare against eight state-of-the-art baselines spanning GNN-based methods DRHGCN (Cai *et al*. 2021), DRWBNCF (Meng *et al*. 2022), and AMDGT (Liu *et al*. 2024a), and GCL-based methods SADR (Jin *et al*. 2024), SGCD (Yu *et al*. 2025), DRGCL (Jia *et al*. 2025), MICLE (Cui *et al*. 2025), and DRMAHGC (Liu *et al*. 2024b). Detailed dataset statistics and baseline descriptions are provided in Supplementary Appendices B and C, available as supplementary data at *Bioinformatics* online, respectively.

### 3.2 Implementation details

Experiments are conducted using 10-fold cross-validation on three benchmark datasets. The ratio of positive to negative samples is set to 1:1; imbalance experiments are provided in Supplementary Appendix I, available as supplementary data at *Bioinformatics* online, and cold-start/inductive validations are provided in Supplementary Appendix J, available as supplementary data at *Bioinformatics* online. For spectral encoding, the enhancement ratio β is selected from [0.1, 1.0], and the retained frequency number *K* is adaptively determined by graph size and bounded by $K_{\text{min}}$ and $K_{\text{max}}$, with detailed settings provided in Supplementary Appendix E, available as supplementary data at *Bioinformatics* online. Wave evolution learning parameters include time parameter *T* selected from {0.5,0.8,1.0,1.5,2.0} and time step Δt selected from {0.2,0.4,0.6,0.8,1.0}. The heterogeneous semantic encoder uses 1 layer with 4 attention heads, while the homogeneous similarity encoder uses 2 layers with 4 attention heads, both with output dimension 64. The MLP predictor consists of 4 layers with dimensions [64, 1024, 1024, 256, 2] and a dropout rate of 0.4. Training employs the Adam optimizer with learning rate $1\times 10^{-3}$, weight decay $1\times 10^{-5}$, and 200 epochs. The loss balancing coefficient α is selected from {0.3,0.5,0.7}, temperature parameters $\tau^{\mathrm{cross}}$, $\tau^{\mathrm{intra}}$ from {0.5,0.8,1.0}, and contrastive loss weights $\lambda^{\mathrm{cross}}$, $\lambda^{\mathrm{intra}}$ from {0.3,0.5,0.7}. The dropout rate for all encoders is set to 0.2.

### 3.3 Drug-disease link prediction

To evaluate the effectiveness of the proposed method for drug repositioning, we conduct drug-disease association prediction experiments on three public benchmark datasets using 10-fold cross-validation, comparing against eight representative baseline methods. Table 1 presents the performance comparison results. The proposed method achieves the highest AUC, AUPR, recall, accuracy, and F1 scores on all three datasets, while remaining competitive in precision. On B-dataset, AUC and AUPR reach 0.9953 and 0.9940, respectively, representing improvements of 2.05 and 2.18 percentage points over the second-best method DRMAHGC (0.9748 and 0.9722). Similar advantages are maintained on C-dataset and F-dataset, with AUC exceeding 0.994 on both. Notably, recall approaches or exceeds 0.99 across all datasets, indicating that the model can effectively identify nearly all potential therapeutic associations, which is crucial for discovering new repositioning opportunities. Compared to spatial graph neural networks (DRGCL, SGCD), spectral global modeling more effectively captures long-range transitive associations and multi-step action mechanisms. Compared to heterogeneous graph methods (DRHGCN, MICLE), wave evolution maintains discriminative ability among drug candidates while mining indirect associations, avoiding over-smoothing. The method maintains consistently excellent performance across datasets of different scales and characteristics, with standard deviations generally below 0.01, demonstrating robustness and generalization capability. This validates the effectiveness of the spatial-spectral collaborative framework, where multi-scale similarity propagation captured by wave evolution and long-range biological mechanisms captured spectrally provide complementary evidence for drug repositioning prediction. Additional score-distribution analysis and a fold-wise graphon-augmentation audit are provided in Supplementary Appendix K, available as supplementary data at *Bioinformatics* online.

**Table 1:** Drug-disease link prediction results on B-dataset, C-dataset, and F-dataset.

Dataset	Model	AUC	AUPR	Recall	Precision	Accuracy	F1
B-dataset	SADR	0.9211±0.004	0.9209±0.003	0.8559±0.004	0.8512±0.003	0.8525±0.005	0.8548±0.003
	DRGCL	0.9247±0.003	0.9230±0.005	0.8626±0.007	0.8544±0.008	0.8546±0.006	0.8582±0.006
	SGCD	0.9235±0.003	0.9224±0.002	0.8631±0.003	0.8458±0.005	0.8506±0.004	0.8536±0.003
	AMDGT	0.9317±0.002	0.9302±0.003	0.8619±0.002	0.8619±0.002	0.8593±0.002	0.8616±0.003
	DRHGCN	0.9092±0.002	0.9106±0.002	0.7711±0.001	0.8678±0.001	0.8368±0.002	0.8166±0.001
	DRWBNCF	0.9004±0.001	0.9018±0.002	0.2021±0.004	**0.9810** ± **0.002**	0.5991±0.002	0.3352±0.003
	MICLE	0.9306±0.003	0.9300±0.003	0.8686 ± 0.007	0.8537±0.005	0.8599±0.005	0.8611±0.005
	DRMAHGC	0.9748 ± 0.003	0.9722 ± 0.004	0.8653±0.005	0.9166±0.018	0.8933 ± 0.011	0.8902 ± 0.009
	Ours	**0.9953** ± **0.002**	**0.9940** ± **0.003**	**0.9972** ± **0.003**	0.9374 ± 0.015	**0.9652** ± **0.008**	**0.9663** ± **0.008**
C-dataset	SADR	0.9550±0.005	0.9584±0.003	0.8853±0.004	0.8768±0.003	0.8994±0.005	0.8806±0.004
	DRGCL	0.9606±0.003	0.9619±0.004	0.9045±0.006	0.8988±0.005	0.9041±0.006	0.9013±0.008
	SGCD	0.9564±0.004	0.9555±0.006	0.8717±0.003	0.8658±0.005	0.8974±0.003	0.8695±0.003
	AMDGT	0.9672±0.003	0.9696±0.003	0.9250±0.004	0.8768±0.003	0.9052±0.003	0.9078±0.003
	DRHGCN	0.9324±0.003	0.9427±0.004	0.8008±0.002	0.9192±0.001	0.8652±0.002	0.8559±0.002
	DRWBNCF	0.9234±0.004	0.9419±0.004	0.8370±0.004	0.8984±0.002	0.8663±0.004	0.8612±0.004
	MICLE	0.9607±0.008	0.9668±0.007	0.9210±0.020	0.9086±0.010	0.9141±0.010	0.9146±0.010
	DRMAHGC	0.9827 ± 0.007	0.9812 ± 0.008	0.9505 ± 0.017	**0.9460** ± **0.018**	0.9480 ± 0.013	0.9481 ± 0.013
	Ours	**0.9952** ± **0.003**	**0.9942** ± **0.004**	**0.9964** ± **0.003**	0.9253 ± 0.020	**0.9578** ± **0.011**	**0.9595** ± **0.011**
F-dataset	SADR	0.9504±0.006	0.9578±0.005	0.9123±0.004	0.8719±0.005	0.8914±0.003	0.8916±0.005
	DRGCL	0.9525±0.005	0.9602±0.004	0.9152±0.006	0.8879±0.008	0.8985±0.007	0.9005±0.008
	SGCD	0.9496±0.003	0.9550±0.004	0.9175 ± 0.005	0.8663±0.005	0.8940±0.004	0.8894±0.004
	AMDGT	0.9584±0.005	0.9607±0.003	0.9146±0.003	0.8730±0.003	0.8908±0.003	0.8928±0.005
	DRHGCN	0.9207±0.004	0.9375±0.002	0.7739±0.002	0.9309 ± 0.001	0.8583±0.001	0.8452±0.002
	DRWBNCF	0.8958±0.005	0.9200±0.004	0.8237±0.004	0.8752±0.003	0.8296±0.002	0.8341±0.004
	MICLE	0.9544±0.010	0.9591±0.012	0.9066±0.013	0.9003±0.015	0.9066±0.013	0.9074±0.013
	DRMAHGC	0.9787 ± 0.008	0.9782 ± 0.009	0.9150±0.033	**0.9511** ± **0.022**	0.9336 ± 0.019	0.9322 ± 0.020
	Ours	**0.9945** ± **0.003**	**0.9928** ± **0.005**	**0.9927** ± **0.008**	0.9284±0.021	**0.9578** ± **0.010**	**0.9593** ± **0.009**

Best Results Are in Bold, Second-Best Are Underlined.

### 3.4 Ablation study

To verify the effectiveness of each component in the proposed framework, we conduct ablation experiments. Specifically, “w/o WEL” removes the wave evolution module, “w/o FreqEnco” removes spectral encoding of homogeneous similarity networks, “w/o FreqHenco” removes spectral encoding of heterogeneous biological networks, and “All” represents the complete model. Experiments are evaluated using 10-fold cross-validation on three datasets.

**Figure 2:**
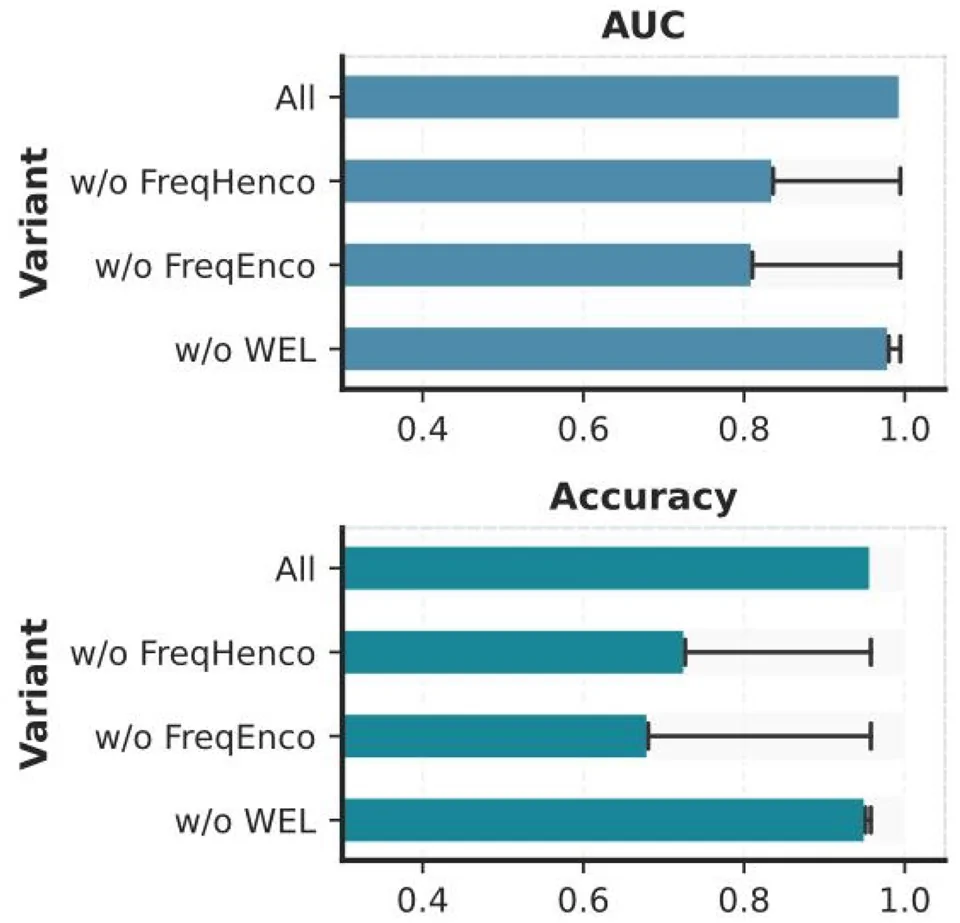
Ablation experiment results on F-Dataset.

The ablation results (Fig. 2) demonstrate that removing spectral encoding of homogeneous similarity networks (w/o FreqEnco) leads to a sharp performance decline, with AUC dropping from approximately 99% to around 80% and recall plummeting from 99% to 40%–50%, validating the critical role of spectral global modeling in capturing long-range transitive associations. Removing spectral encoding of heterogeneous networks (w/o FreqHenco) similarly results in significant performance degradation, indicating that biological mechanism reasoning based on drug-protein-disease networks provides necessary complementary information for association prediction. Removing wave evolution (w/o WEL) causes slight performance decline but still maintains relatively high levels, demonstrating that wave evolution effectively enhances feature representations by capturing multi-scale similarity propagation in the spatial domain and avoids the over-smoothing problem in deep graph neural networks. The experimental results validate the necessity and effectiveness of each component in the spatial-spectral collaborative modeling framework. The spectral encoding modules capture long-range associations difficult to identify by spatial methods through global modeling capability, while wave evolution provides discriminative multi-scale feature representations. The synergistic interaction of these components collectively supports the model’s superior performance in drug repositioning tasks. Complete ablation results on all three datasets and all six evaluation metrics are provided in Supplementary Appendix D, available as supplementary data at *Bioinformatics* online.

### 3.5 Hyperparameter sensitivity analysis

To evaluate the impact of key hyperparameters on model performance, we systematically investigate the sensitivity of wave evolution time step Δt and total evolution time *T*, spectral enhancement ratio β, and learning rate across three datasets.

**Figure 3:**
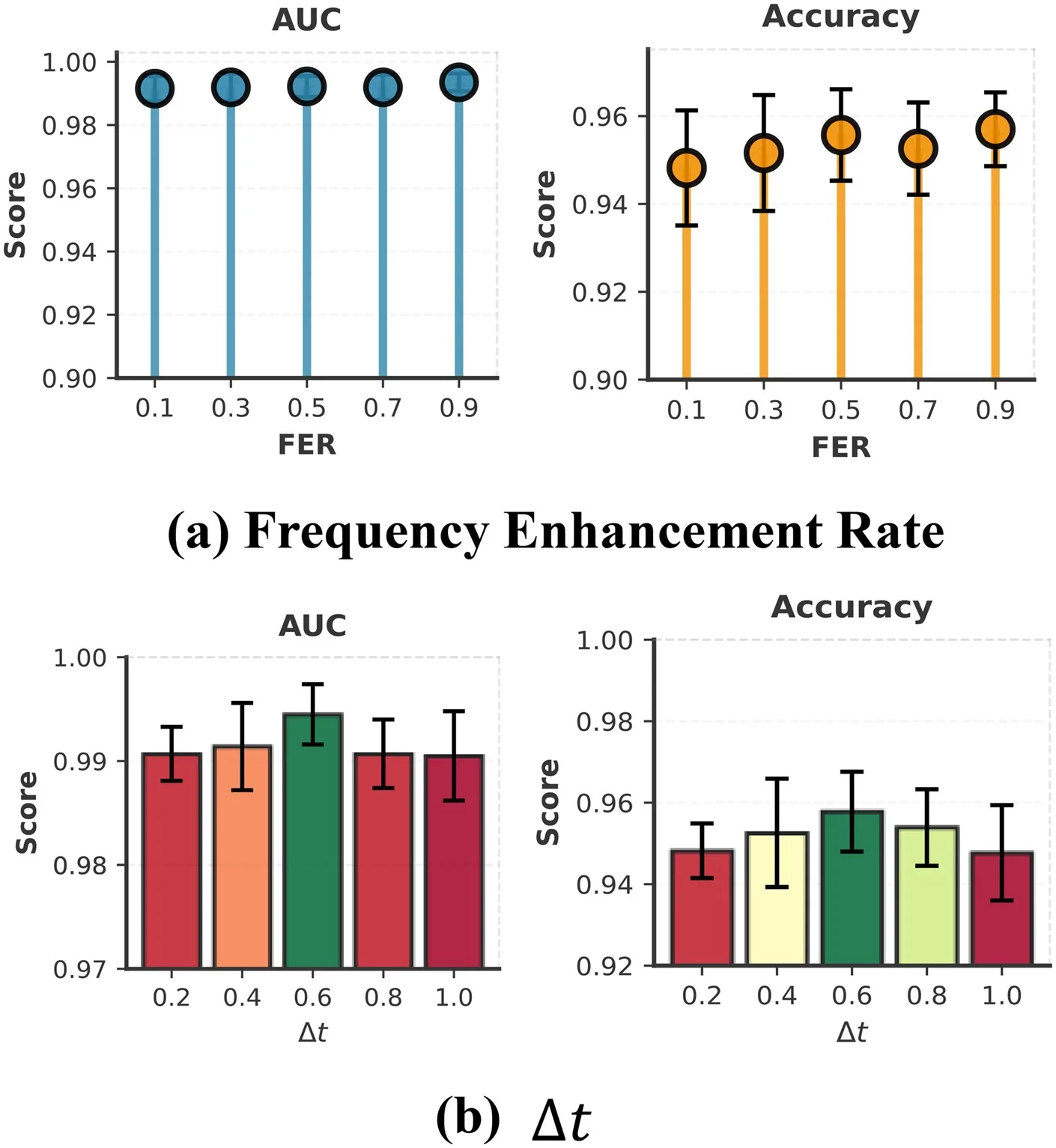
Hyperparameter sensitivity analysis on F-dataset.

**Figure 4:**
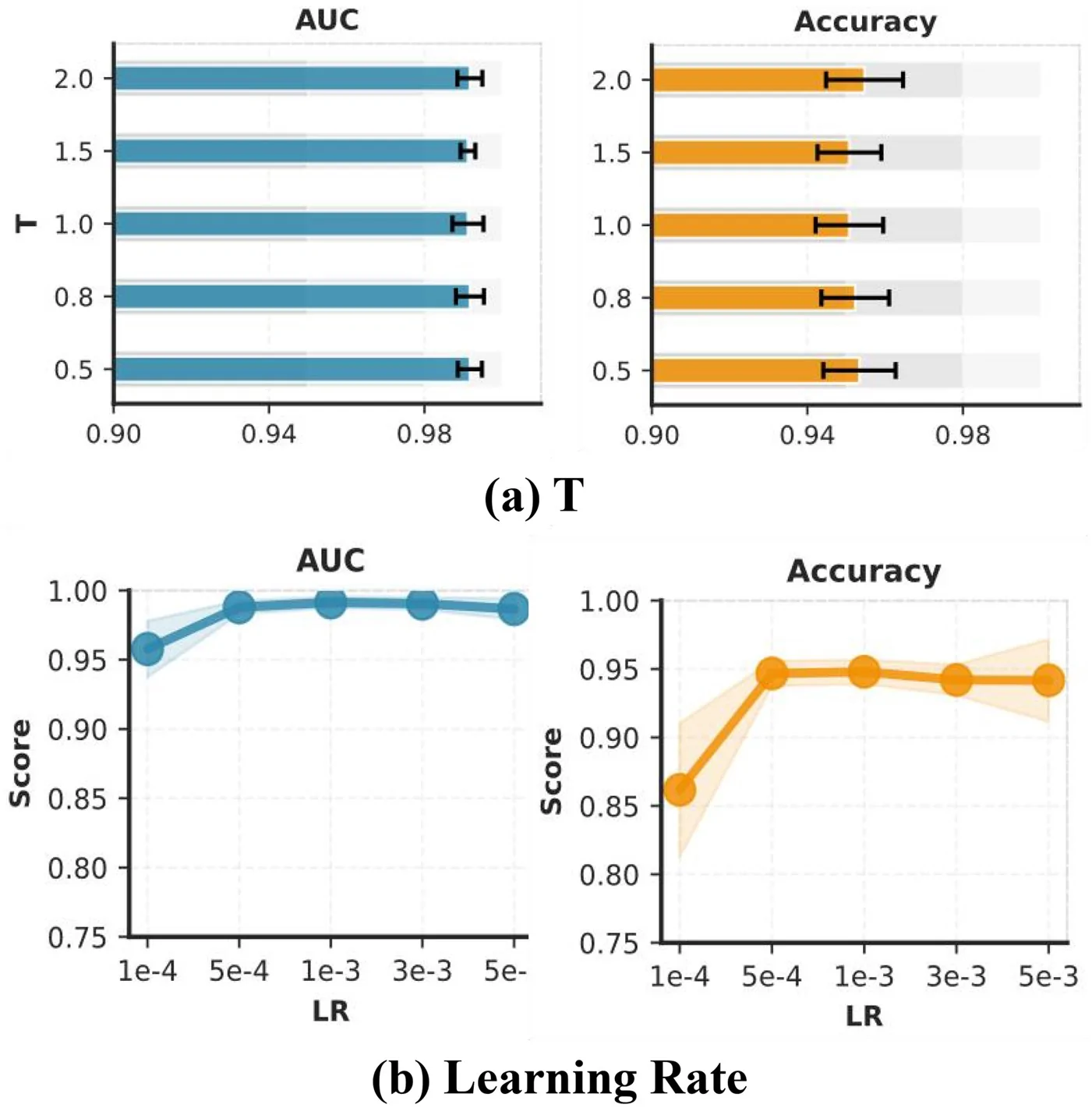
Hyperparameter sensitivity analysis on F-dataset.

The experimental results (Figs. 3 and 4) demonstrate that the model exhibits robust performance with respect to hyperparameters while presenting clear optimal configuration ranges. For wave evolution parameters, time step Δt achieves optimal performance in the range of 0.4–0.6, and total evolution time *T* reaches the best balance at 0.8–1.0, indicating that moderate evolution depth can effectively capture multi-scale similarity propagation without introducing excessive noise. Experiments on spectral enhancement ratio β show that as β increases from 0.1 to 0.9, performance across all three datasets exhibits an upward trend, reaching stable high-performance levels at β≥0.7, validating the critical role of spectral global association modeling in identifying long-range indirect associations. Learning rate experiments indicate that $10^{-3}$ is the optimal choice, achieving a good balance between convergence speed and training stability. Complete hyperparameter sensitivity results on all three datasets and all six evaluation metrics are provided in Supplementary Appendix E, available as supplementary data at *Bioinformatics* online.

### 3.6 Case study

To evaluate the model’s therapeutic candidate discovery ability, we conducted case studies on Alzheimer’s disease and Parkinson’s disease. Representative predicted candidates were manually validated using PubMed-indexed literature and reordered according to evidence strength. Table 2 presents the curated results. Using a broad validation criterion covering clinical, preclinical, cellular, pathway-level, and computational evidence, most Alzheimer’s disease candidates and several Parkinson’s disease candidates were supported by published evidence. For Alzheimer’s disease, Dronabinol showed clinical benefit for agitation, while Meloxicam, Hydralazine, Metformin, and Leucovorin were supported by disease-model or mechanistic studies. Theophylline, Budesonide, Isotretinoin, Azelaic acid, and Dorzolamide were supported by AChE-related, cellular, retinoid-related, neurocognitive, or computational pathway-level evidence. For Parkinson’s disease, Cabergoline had direct clinical relevance, whereas Dexamethasone, Captopril, and Tizanidine showed clinical or model-based evidence. Fluoxetine and Ofloxacin were supported by indirect clinical or broader neurodegenerative evidence, while Amrinone, Adenosine phosphate, Alanine, and Spironolactone lacked acceptable drug-specific Parkinson’s disease evidence. These results demonstrate the model’s ability to recover literature-supported associations and generate biologically plausible candidates requiring further validation.

**Table 2:** Curated drug candidate results for Alzheimer’s disease and Parkinson’s disease.

**Alzheimer’s disease**			
Rank	Drug name	DrugBank ID	PMID
1	Dronabinol	DB00470	41350162
2	Meloxicam	DB00814	36279100
3	Hydralazine	DB01275	37661037
4	Metformin	DB00331	29253574
5	Leucovorin	DB00650	39582067
6	Theophylline	DB00277	38634273
7	Budesonide	DB01222	15854773
8	Isotretinoin	DB00982	15897158
9	Azelaic acid	DB00548	39688203
10	Dorzolamide	DB00869	38002524

**Parkinson’s Disease**			
Rank	Drug name	DrugBank ID	PMID
1	Cabergoline	DB00248	9040722
2	Dexamethasone	DB01234	39672994
3	Captopril	DB01197	24184050
4	Tizanidine	DB00697	18602099
5	Fluoxetine	DB00472	23424961
6	Ofloxacin	DB01165	33132905
7	Amrinone	DB01427	–
8	Adenosine phosphate	DB00131	–
9	Alanine	DB00160	–
10	Spironolactone	DB00421	–

PMID indicates representative clinical, preclinical, cellular, pathway-level, or computational evidence where available.

### 3.7 Molecular docking experiments

**Figure 5:**
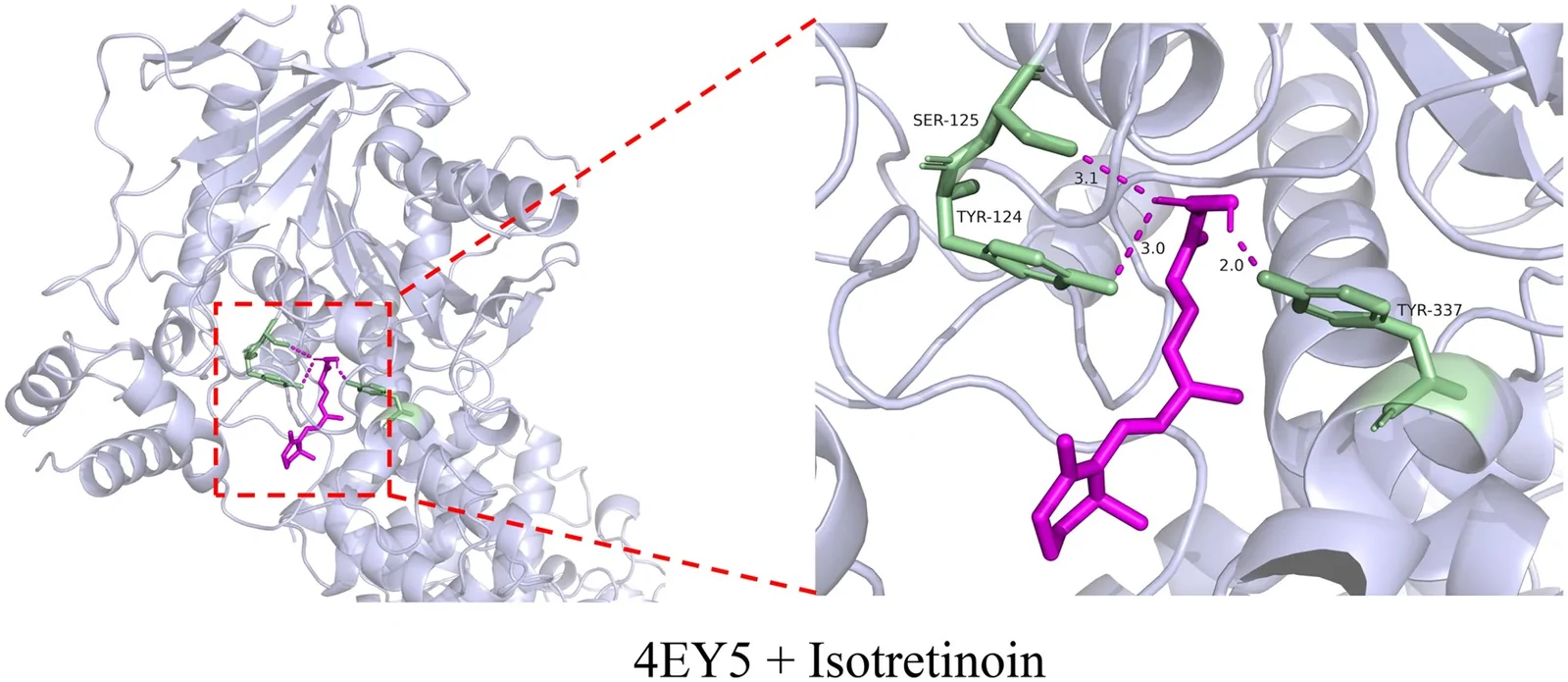
Molecular docking experiment results.

Molecular docking experiments are performed on Isotretinoin and Leucovorin, two predicted Alzheimer’s disease candidates selected for structural validation, with key target proteins 4EY5 and 4XXS using AutoDock. Fig. 5 shows Isotretinoin-4EY5 docking, revealing a plausible binding pose at the active site with hydrogen bond interactions through multiple residues. Additional docking configurations, including Leucovorin-4EY5, Isotretinoin-4XXS, and Leucovorin-4XXS, are provided in Supplementary Appendix F, available as supplementary data at *Bioinformatics* online. All four drug-target pairs yielded negative docking scores, suggesting energetically favourable predicted binding poses. These results provide structural-level support for the predicted associations, indicating that candidates with limited direct clinical evidence may still interact with Alzheimer’s disease-related targets. The findings demonstrate the model’s ability to discover non-explicit therapeutic associations and provide a molecular basis for further validation, highlighting computational methods’ value in guiding drug repositioning research.

## 4 Conclusion

In this study, we proposed a spatial-spectral collaborative framework for drug repositioning that jointly captures multi-scale similarity propagation and long-range heterogeneous biological associations. Experimental results on three benchmark datasets and case studies demonstrate its effectiveness in identifying potential therapeutic associations. The ablation analysis further shows that spectral encoding, especially the homogeneous similarity spectral encoder, plays a central role in capturing global association patterns, while wave evolution provides complementary multi-scale discrimination. Nevertheless, the model may be less informative for completely unseen drugs or diseases when association-derived similarity features are unavailable, and spectral decomposition may introduce additional computational cost on very large graphs. Future work will focus on improving inductive generalization and developing more scalable spectral modeling strategies.

## Supplementary Material

btag553_Supplementary_Data

## Data Availability

The source code and datasets used in this study are available at https://github.com/Juniper-cola/BIO_SSF.
