# Supplementary material for: Spatial-spectral fusion enables drug repositioning by capturing indirect and long-range associations in biological networks
Source: Bioinformatics. 2026 Aug 10;42(8):btag553. doi: 10.1093/bioinformatics/btag553 (PMC13461371; doi:10.1093/bioinformatics/btag553)
Supplement: Supplementary file 1 [file btag553_supplementary_data.pdf]

Supplementary Material

Appendix A
Related Work

A. Computational Drug Repositioning

Computational drug repositioning methods have evolved from shallow models to deep graph-based approaches. Early methods such as DTINet [1] and DeepDR [2] rely on matrix factorization or shallow autoencoders to exploit direct similarity relationships, but cannot capture indirect therapeutic associations beyond first-order proximity. GNN-based methods advance this direction by learning from graph topology. LAGCN [3] introduces layer attention to fuse multi-scale GCN features, HDGAT [4] models hierarchical drug-disease interactions, DRHGCN [5] applies inter- and intra-domain graph convolutions, and DRWBNCF [6] integrates bilinear interactions with neighborhood information. AMDGT [7] further combines multi-modal biochemical data through dual graph transformers. However, stacking GNN layers to expand receptive fields inevitably leads to over-smoothing [8], where node representations converge and lose discriminative power in highly connected biological networks. Heterogeneous graph methods such as HINGRL [9], RLFDDA [10], SFRLDDA [11], and GDLDR [12] propagate information across drug-protein-disease networks, yet remain constrained by the local neighborhood aggregation paradigm that expands receptive fields by only one hop per layer. These fundamental limitations motivate our spatial-spectral framework that bypasses locality constraints through spectral global modeling.

B. Spectral Methods on Graphs

Spectral graph theory provides a principled foundation for analyzing graph-structured data through the eigendecomposition of graph Laplacians [8]. Classical spectral methods, including ChebNet and GCN, approximate spectral filters with polynomials of the Laplacian, enabling localized filtering in the vertex domain. Recent work has demonstrated the advantages of spectral approaches for capturing global structural patterns: low-frequency eigenvectors encode community-level structure while high-frequency components capture local variations. In the biomedical domain, Zhang *et al.* [13] introduced a structure-preserving spectral expansion module that injects global topology from biomedical knowledge graphs via spectral coordinates, achieving state-of-the-art performance on drug-kinase interaction prediction. However, existing spectral methods are primarily designed for homogeneous graphs and lack customized modules for heterogeneous biological networks involving multiple entity types and relation types. Our framework addresses this gap by designing network-specific spectral encoding modules for both homogeneous similarity networks and heterogeneous drug-protein-disease networks, enabling direct modeling of long-range associations across different biological entities.

C. Graph Contrastive Learning

Graph contrastive learning has emerged as an effective paradigm for alleviating data sparsity in biological network analysis. SADR [14] combines data augmentation with adaptive denoising to learn robust node representations. SGCD [15] performs semantic-enriched augmentation by mining potential neighborhood relationships in embedding space. DRGCL [16] constructs semantic graphs from top-K similar neighbors to enhance embedding consistency between topological and semantic views. MICLE [17] designs complementary inter-view and intra-view contrastive tasks on heterogeneous biological networks without stochastic perturbation. DRMAHGC [18] combines graph-masked autoencoders with heterogeneous graph contrastive learning using structure- and metapath-level augmentation based on graphon estimation [19]. Despite these advances, existing methods construct contrastive views primarily through random perturbation or augmentation strategies, lacking explicit alignment between similarity-based and mechanism-based reasoning. Our framework introduces a dual-view contrastive learning design that systematically aligns representations from homogeneous similarity networks and heterogeneous biological mechanism networks, providing complementary evidence for drug repositioning prediction.

Appendix B
Dataset Details

This appendix supplements the datasets and baseline methods subsection in the main text by providing detailed statistics for B-dataset, C-dataset, and F-dataset.

We evaluate the proposed method on three benchmark datasets from the drug repositioning domain: B-dataset [20], C-dataset [21], and F-dataset [22]. Each dataset consists of three biological entities (drugs, diseases, proteins) connected by three relationship types: drug-disease associations (DDA), drug-protein interactions (DRP), and disease-protein connections (DIP).

B-dataset: Drug-disease associations are extracted from the Comparative Toxicogenomics Database (CTD), covering conditions such as atrioventricular block, glomerulonephritis, and hyperlipidemia. Disease categorization follows the MEDIC-Slim taxonomy based on MeSH hierarchy. The dataset contains 269 drugs, 598 diseases, and 1,021 proteins, with 18,416 drug-disease associations, 3,110 drug-protein interactions, and 5,898 disease-protein connections, yielding an association density of 11.45%.

C-dataset: Drugs and diseases are sourced from DrugBank and OMIM databases, respectively. This dataset exhibits diverse disease granularity, ranging from common cancers to rare genetic disorders like encephalopathy with necrotizing myopathy. It comprises 663 drugs, 409 diseases, and 993

proteins, with 2,532 drug-disease associations, 3,672 drug-protein interactions, and 10,691 disease-protein relationships, with an association density of 0.93%.

F-dataset: Drug and disease data are obtained from DrugBank and OMIM databases. As the largest protein repository among the three datasets, F-dataset contains 592 drugs, 313 diseases, and 2,741 proteins, with drug-protein associations from DrugBank and disease-protein associations from DisGeNET. The dataset includes 1,933 drug-disease associations, 3,152 drug-protein interactions, and 47,470 disease-protein relationships, with an association density of 1.04%.

The lower association densities in C-dataset and F-dataset create more data-scarce evaluation scenarios, which are important for assessing model performance in real-world drug repositioning applications where known associations are limited.

Appendix C
Baseline Methods

This appendix supplements the datasets and baseline methods subsection in the main text by describing the compared methods.

We compare against eight state-of-the-art methods categorized into two groups based on their technical approaches: (1) GNN-based methods that leverage graph neural networks for feature extraction, and (2) GCL-based methods that incorporate graph contrastive learning strategies.

A. GNN-based Methods

DRHGCN designs inter- and intra-domain feature extraction modules by applying graph convolution operations on drug similarity, disease similarity, and drug-disease association networks. It introduces a layer attention mechanism to fuse multi-layer GCN outputs for improved prediction performance.

DRWBNCF proposes a weighted bilinear graph convolution operation to integrate known drug-disease associations, neighborhood information, and neighborhood interactions into a unified representation. The method employs an MLP optimized with α-balanced focal loss and graph regularization to handle class imbalance.

AMDGT constructs a multi-modal framework based on dual graph transformers, combining similarity data with biochemical information through an attention-aware modality interaction architecture for effective multi-modal feature fusion.

B. GCL-based Methods

SADR employs data augmentation and contrastive learning strategies to learn node representations for alleviating data sparsity. It incorporates an adaptive denoising training component to identify and mitigate noise during the training process.

SGCD performs semantic-enriched augmentation from the embedding layer perspective, mining potential neighborhood relationships in semantic space and combining similar drugs as prototype contrastive targets with adaptive denoising mechanisms.

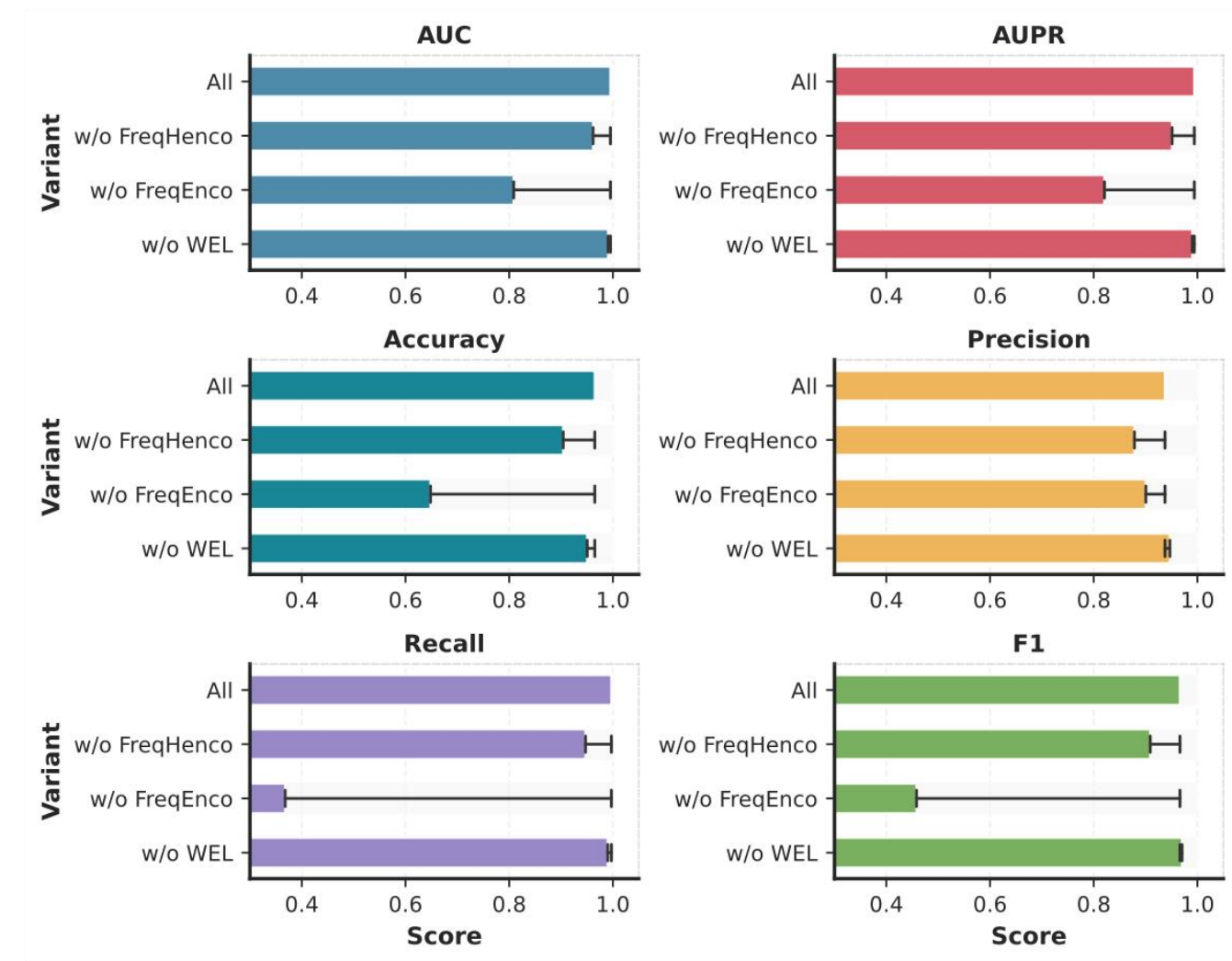

Ablation experimental results on B-dataset

Fig. S1. Complete ablation experimental results on B-dataset across all six evaluation metrics. The results demonstrate consistent effectiveness of each component.

DRGCL constructs semantic graphs by selecting top-K similar neighbors from drug/disease similarity information instead of random augmentation. It enhances embedding consistency between topological and semantic features through graph contrastive learning.

MICLE constructs a heterogeneous biological network by integrating drug-protein and disease-protein bipartite graphs, and employs heterogeneous graph transformer to capture rich connectivity patterns. It designs complementary inter-view and intra-view contrastive learning tasks without relying on stochastic perturbation augmentation.

DRMAHGC generates high-order features from similarity networks using a graph-masked autoencoder, and employs heterogeneous graph contrastive learning with structure- and metapath-level augmentation. A positive-fusion negative sampling strategy is exploited to synthesize informative negative samples for training.

Appendix D
Complete Ablation Results

This appendix supplements the ablation study in the main text by providing complete results across all six evaluation metrics on all three benchmark datasets.

To comprehensively validate the contribution of each component across all benchmark datasets, we conduct ablation experiments on B-dataset, C-dataset, and F-dataset with complete evaluation metrics. Specifically, "w/o WEL" removes the spatial wave evolution module, "w/o FreqEnco" removes spectral encoding for homogeneous similarity networks, "w/o FreqHenco" removes spectral encoding for heterogeneous biological networks, and "All" denotes the complete model. While the main text summarizes the F-dataset ablation results, here we provide comprehensive results across all six evaluation metrics (AUC, AUPR, Recall, Precision, Accuracy, F1) for all three datasets.

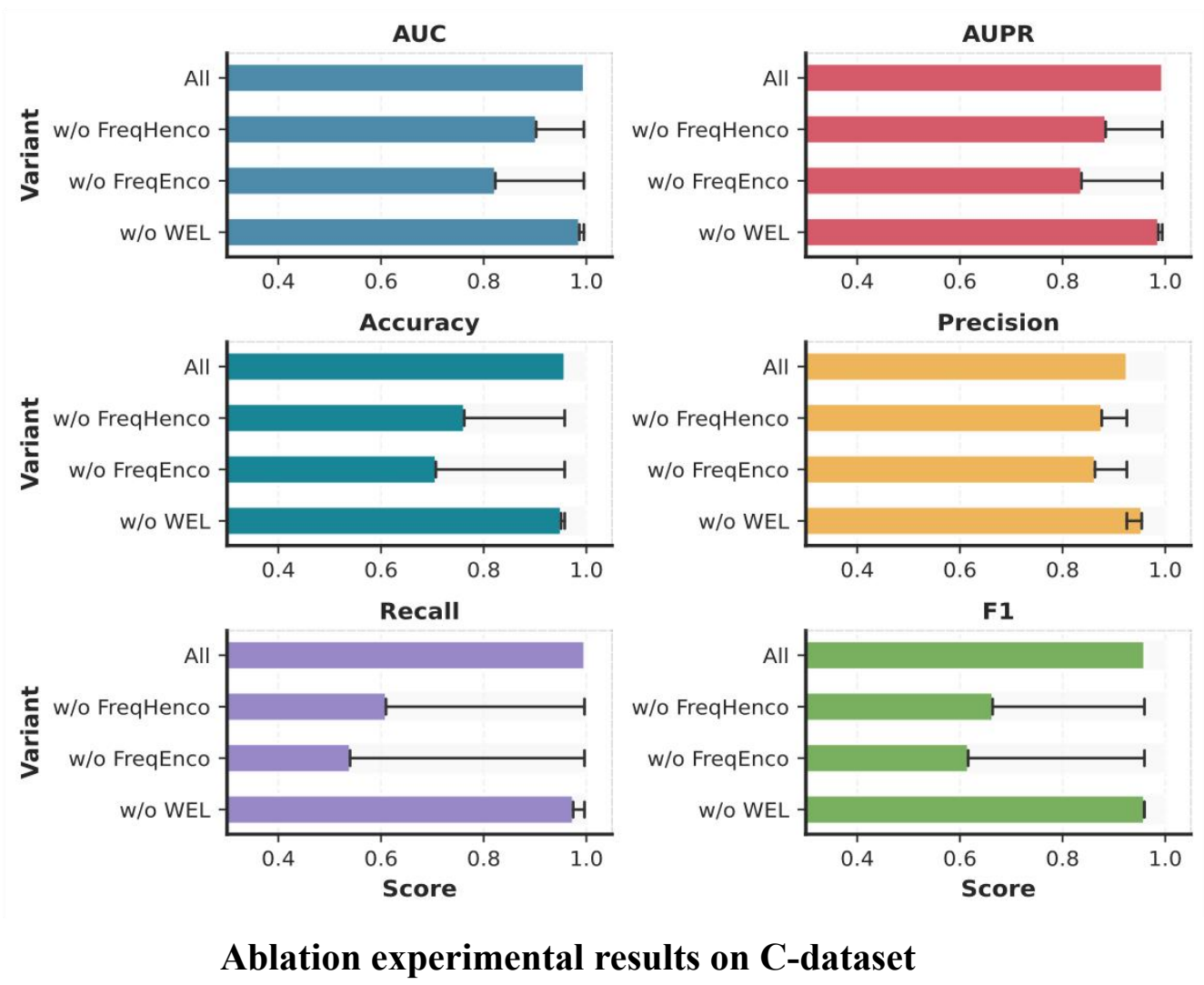

Fig. S2. Complete ablation experimental results on C-dataset across all six evaluation metrics. The consistency with B-dataset results demonstrates model robustness.

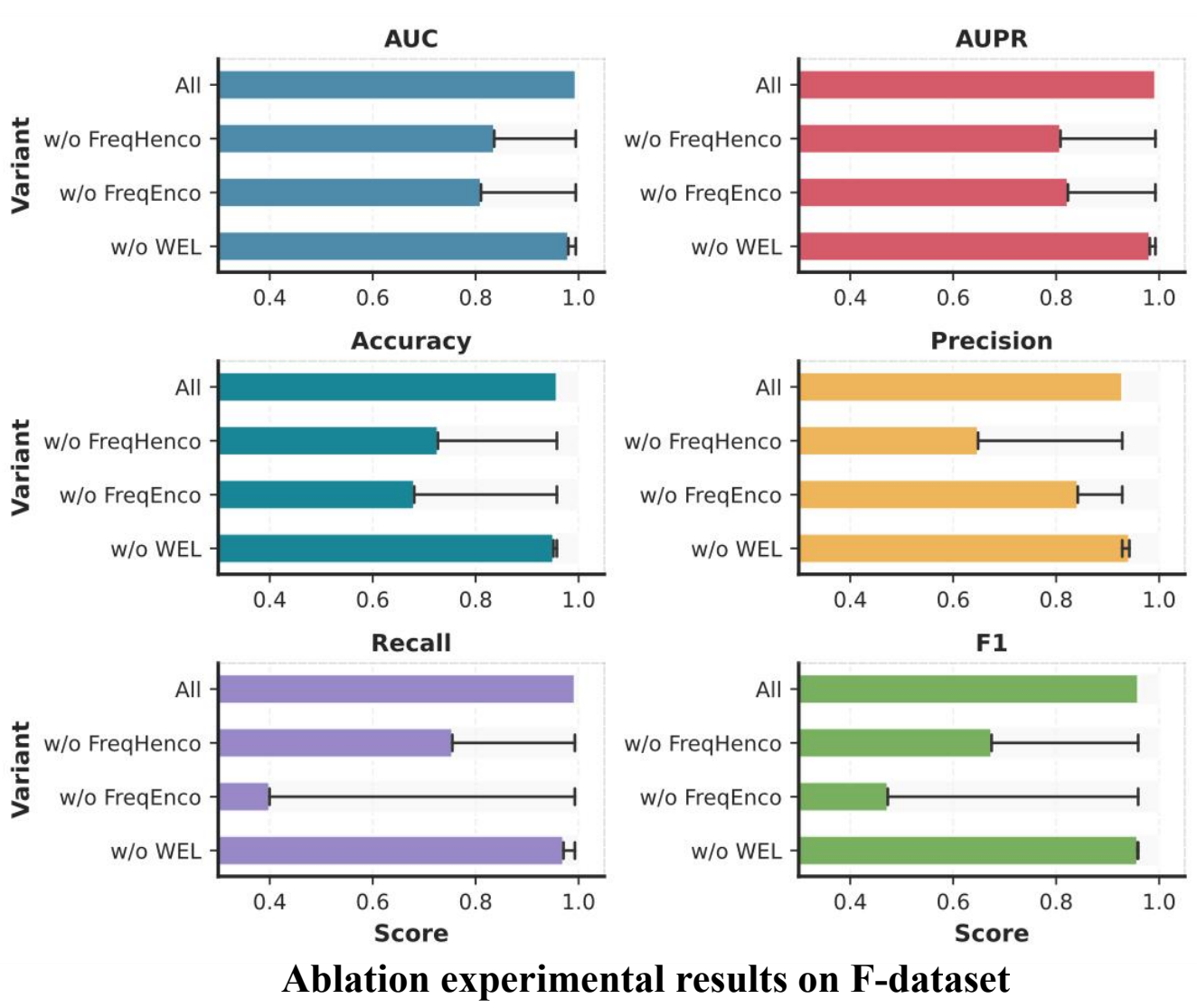

Fig. S3. Complete ablation experimental results on F-dataset across all six evaluation metrics. This complements the main text by including Recall, Precision, Accuracy, and F1 scores.

A. B-dataset Ablation Study

Fig. S1 presents the complete ablation results on B-dataset across all six metrics. The results demonstrate that removing spectral encoding for homogeneous similarity networks (w/o FreqEnco) causes substantial performance degradation across all metrics. AUC drops from approximately 99% to around 80%, and Recall plummets from 99% to 40%–50%, confirming the critical role of spectral global modeling in capturing long-range transitive associations. Removing spectral encoding for heterogeneous networks (w/o FreqHenco) also yields significant performance decline, validating that drug-protein-disease network reasoning provides essential complementary biological information. Removing spatial wave evolution (w/o WEL) leads to marginal performance reduction while maintaining relatively high levels, demonstrating that wave-based spatial propagation effectively enhances feature representations while avoiding over-smoothing.

B. C-dataset Ablation Study

Fig. S2 presents the complete ablation results on C-dataset. Despite C-dataset having the lowest association density (0.93%) among the three datasets, the ablation patterns remain remarkably consistent with B-dataset findings. The removal of spectral encoding modules (both FreqEnco and FreqHenco) leads to dramatic performance drops across all metrics, while removal of wave evolution causes only modest degradation. This consistency across datasets with vastly different characteristics (11.45% vs 0.93% density) strongly validates the robustness and universality of the spatial-spectral collaborative framework.

C. F-dataset Ablation Study

Fig. S3 presents the complete ablation results on F-dataset across all six evaluation metrics. While the main text shows F-dataset ablation results, here we provide the complete picture including Recall, Precision, Accuracy, and F1 scores. F-dataset, with its large protein repository (2,741 proteins) and moderate sparsity (1.04%), provides a different evaluation scenario. The results maintain the same patterns observed on B and C datasets: spectral encoding modules are essential for high performance (particularly FreqEnco for capturing long-range transitive associations), while spatial wave evolution provides consistent but more modest improvements. The comprehensive six-metric evaluation confirms that all components contribute synergistically to the model's superior performance.

Appendix E
Complete Hyperparameter Sensitivity Analysis

This appendix supplements the hyperparameter sensitivity analysis in the main text by providing complete results across all six evaluation metrics on all three benchmark datasets. We systematically investigate the sensitivity of key hyperparameters across all three benchmark datasets to provide comprehensive evidence of model robustness. The analyzed hyperparameters include spatial wave evolution step size Δt, total propagation depth T, spectral enhancement ratio β, and learning rate. While the main text summarizes F-dataset hyperparameter analysis, here we provide complete results across all six evaluation metrics for all three datasets.

The number of retained frequency components K is not fixed, but is adaptively determined according to the size of the graph used by each spectral encoding module:

$$K = \max\left\{K_{\min}, \min\left(K_{\max}, \lfloor |V| \cdot \rho_{\text{freq}} \rfloor\right)\right\},$$

where $|V|$ denotes the number of nodes in the corresponding graph, ρ_{freq} is the retained low-frequency ratio, and $K_{\min}$ and $K_{\max}$ are the lower and upper bounds of the frequency range, respectively. In this study, we set $\rho_{\text{freq}} = 0.25$, $K_{\min} = 32$, and $K_{\max} = 128$. Thus, the model first selects low-frequency

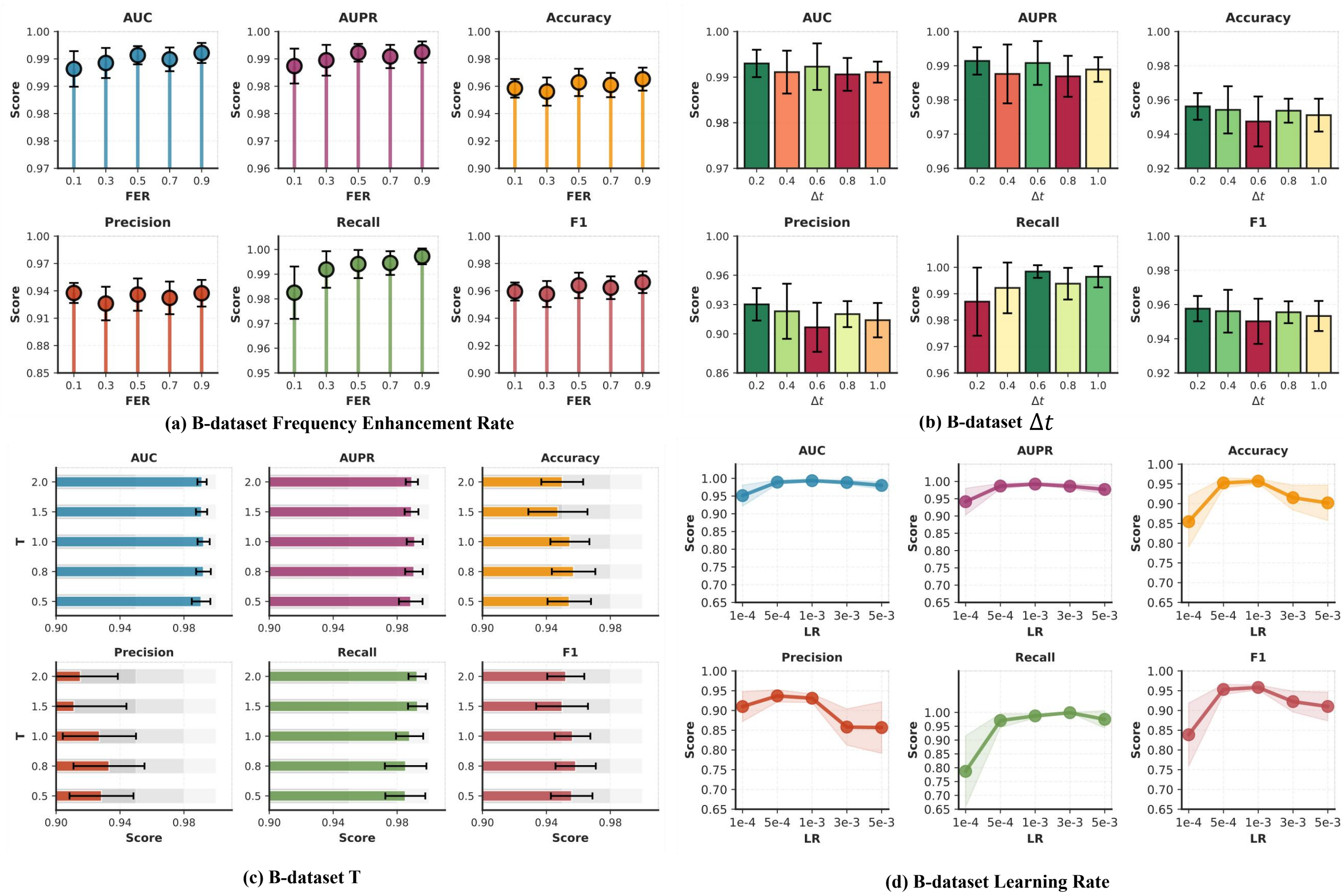

Fig. S4. Complete hyperparameter sensitivity analysis on B-dataset. (a) Spectral enhancement ratio β, (b) Step size Δt, (c) Total propagation depth T, (d) Learning rate. All six evaluation metrics are shown.

components according to a fixed proportion of the graph size, and then clips the value into the range $[32, 128]$. This strategy ensures that small graphs retain sufficient global structural information while preventing large graphs from introducing excessive high-frequency noise. The number of attention heads in each spectral module is set to $H = 4$.

A. B-dataset Hyperparameter Sensitivity

Fig. S4 presents the comprehensive hyperparameter sensitivity results on B-dataset across all six metrics. For spectral enhancement ratio β, performance exhibits a consistent upward trend as β increases from 0.1 to 0.9 across all metrics, reaching stable high-performance levels at $\beta \geq 0.7$. This validates that spectral global association modeling plays a critical role in identifying long-range indirect associations. For spatial wave evolution parameters, step size Δt achieves optimal performance in the range of 0.4–0.6, and total propagation depth T reaches the best balance at 0.8–1.0, indicating that moderate propagation depth effectively captures multi-scale similarity propagation without introducing excessive noise. The learning rate experiments show that 10^{-3} achieves the optimal balance between convergence speed and training stability across all metrics.

B. C-dataset Hyperparameter Sensitivity

Fig. S5 presents the comprehensive hyperparameter sensitivity results on C-dataset. The results demonstrate remarkable consistency with B-dataset findings across all six metrics, despite the significant difference in dataset characteristics. For spectral enhancement ratio β, performance improvements follow a similar upward trend, reaching stable optimal levels at $\beta \geq 0.7$ across all metrics. This consistency across datasets with vastly different sparsity levels (11.45% vs 0.93%) strongly validates that spectral modeling is robust and essential for capturing global association patterns regardless of data availability. Spatial wave evolution parameters show similar optimal ranges: $\Delta t \in [0.4, 0.6]$ and $T \in [0.8, 1.0]$. The learning rate analysis also confirms that 10^{-3} is the optimal choice. The consistency of optimal hyperparameter ranges across all metrics and datasets demonstrates the model's robustness.

C. F-dataset Hyperparameter Sensitivity

Fig. S6 presents the comprehensive hyperparameter sensitivity results on F-dataset across all six evaluation metrics. While the main text shows F-dataset hyperparameter analysis, here we provide the complete picture including Recall, Precision, Accuracy, and F1 scores. The results maintain remarkable consistency with B and C datasets across all metrics. The

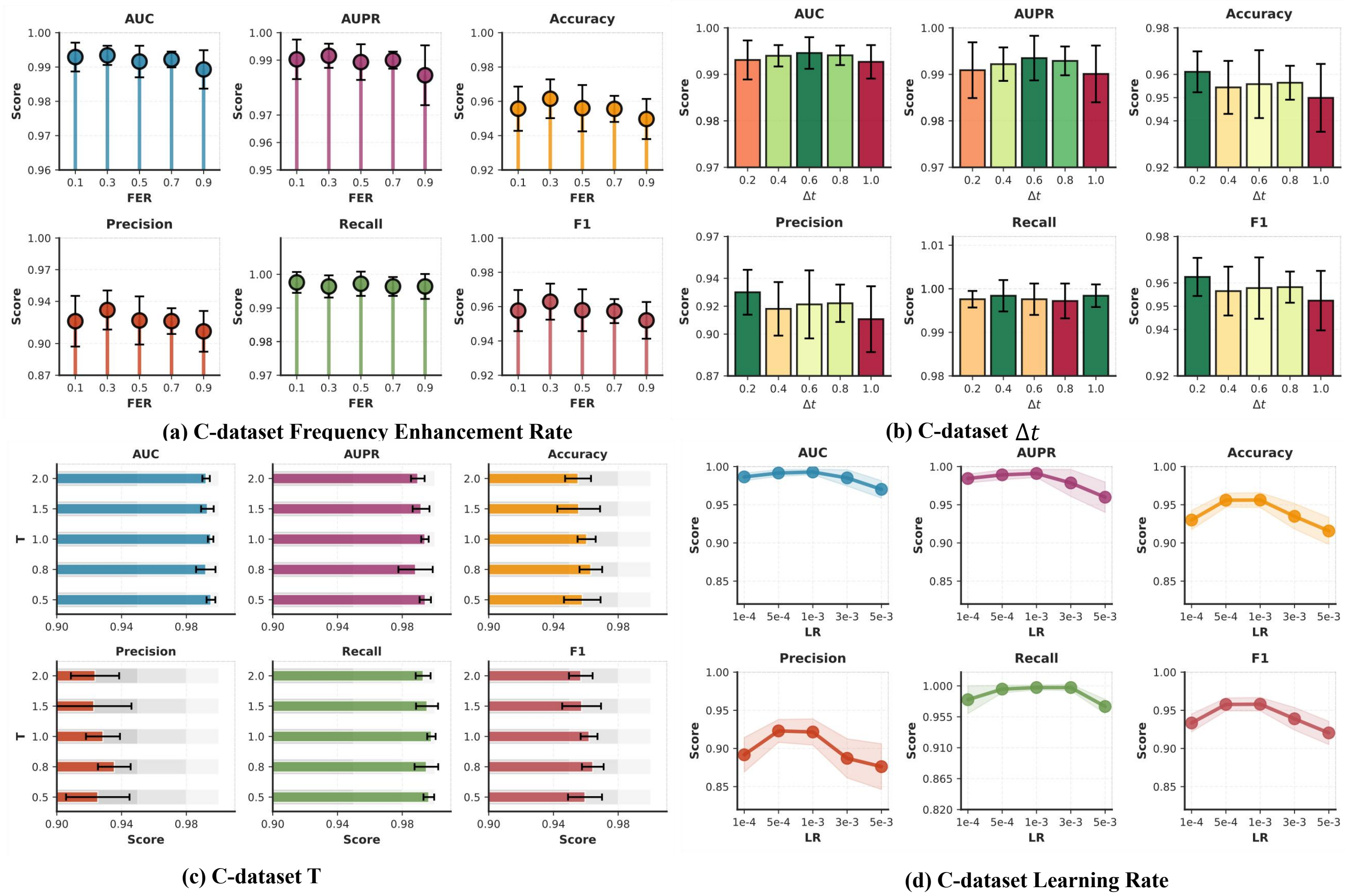

Fig. S5. Complete hyperparameter sensitivity analysis on C-dataset. (a) Spectral enhancement ratio β, (b) Step size Δt, (c) Total propagation depth T, (d) Learning rate. All six evaluation metrics are shown.

spectral enhancement ratio β shows similar upward trends and stabilization at $\beta \geq 0.7$ for all six metrics. Spatial wave evolution parameters (Δt and T) exhibit the same optimal ranges [0.4, 0.6] and [0.8, 1.0] respectively. The learning rate 10^{-3} remains optimal across all metrics. This three-dataset consistency across six metrics provides strong evidence that the proposed framework has minimal hyperparameter sensitivity and excellent generalization capability across different data characteristics.

Appendix F
Additional Molecular Docking Results

This appendix supplements the molecular docking experiments in the main text by presenting three additional drug–target docking configurations that complement the Isotretinoin–4EY5 result: Leucovorin–4EY5, Isotretinoin–4XXS, and Leucovorin–4XXS.

Fig. S7 presents the predicted binding poses and the principal hydrogen-bond and hydrophobic contacts identified by AutoDock. These predicted poses suggest potential interactions between the selected compounds and the examined protein structures.

All four drug–target combinations yielded negative docking scores, suggesting energetically favorable predicted binding poses. However, docking scores do not directly represent experimental binding free energies or establish complex stability. These in silico results provide preliminary structural hypotheses for potential compound–target interactions, which require further experimental validation.

Appendix G
Visualization and Interpretability Analysis

This appendix consolidates the 2D and 3D t-SNE visualization analyses to provide a compact interpretation of the learned representations. Fig. S8 and Fig. S9 show the evolution of node representations across four stages: Original Features, WEL Features after spatial wave evolution learning, FreqEnco after spectral encoding on homogeneous similarity networks, and FreqHenco after spectral encoding on heterogeneous biological networks.

In the original feature space, drugs, diseases, and proteins exhibit substantial overlap with no clear clustering structure, indicating that raw molecular and biological features alone are insufficient for discriminating therapeutic associations. After spatial wave evolution learning, drug and disease nodes begin to aggregate toward their respective regions, while protein nodes form a more structured distribution. This suggests that multi-scale propagation through wave evolution effectively in-

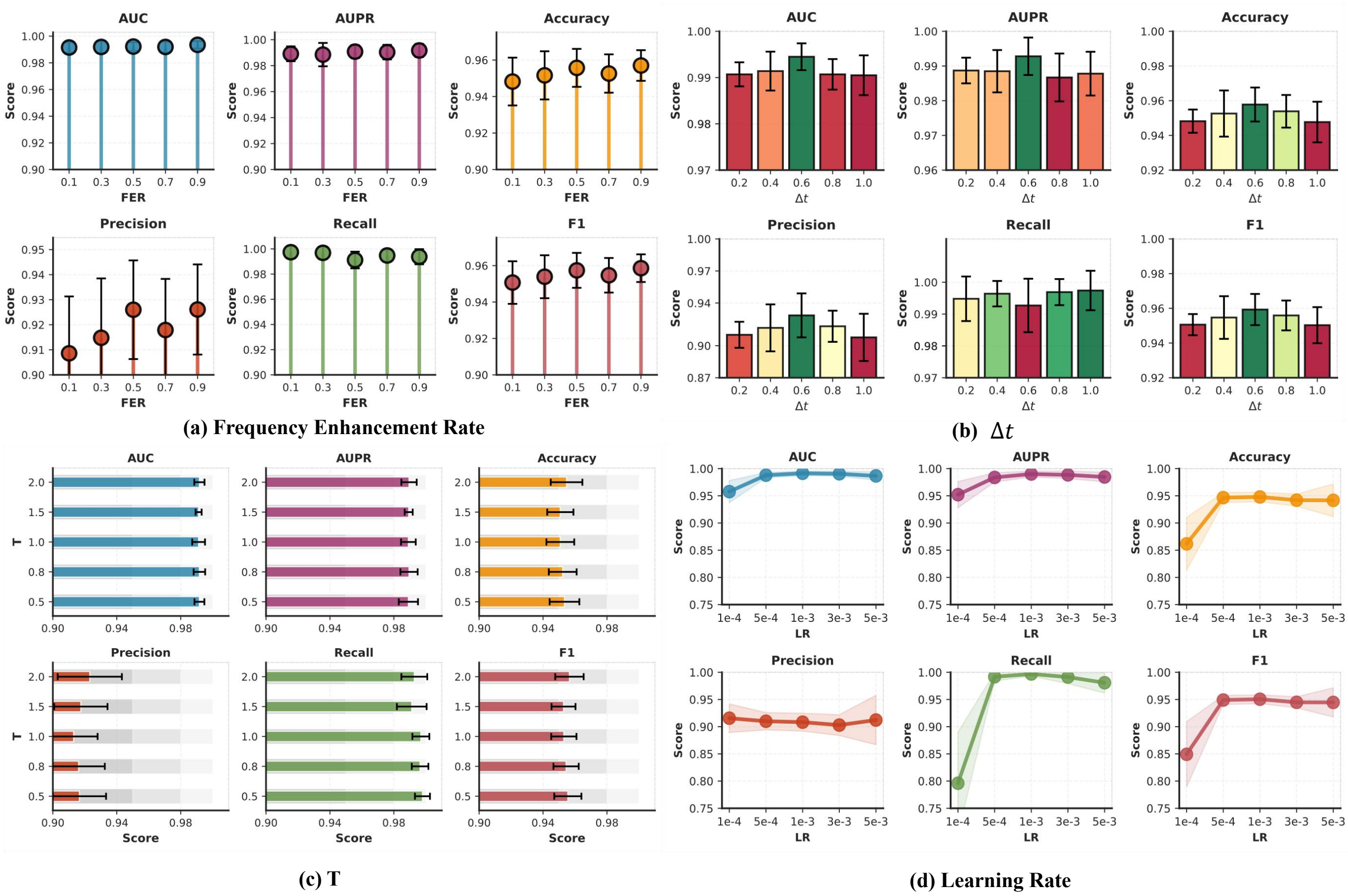

Fig. S6. Complete hyperparameter sensitivity analysis on F-dataset. (a) Spectral enhancement ratio β, (b) Step size Δt, (c) Total propagation depth T, (d) Learning rate. All six evaluation metrics are shown, complementing the main text.

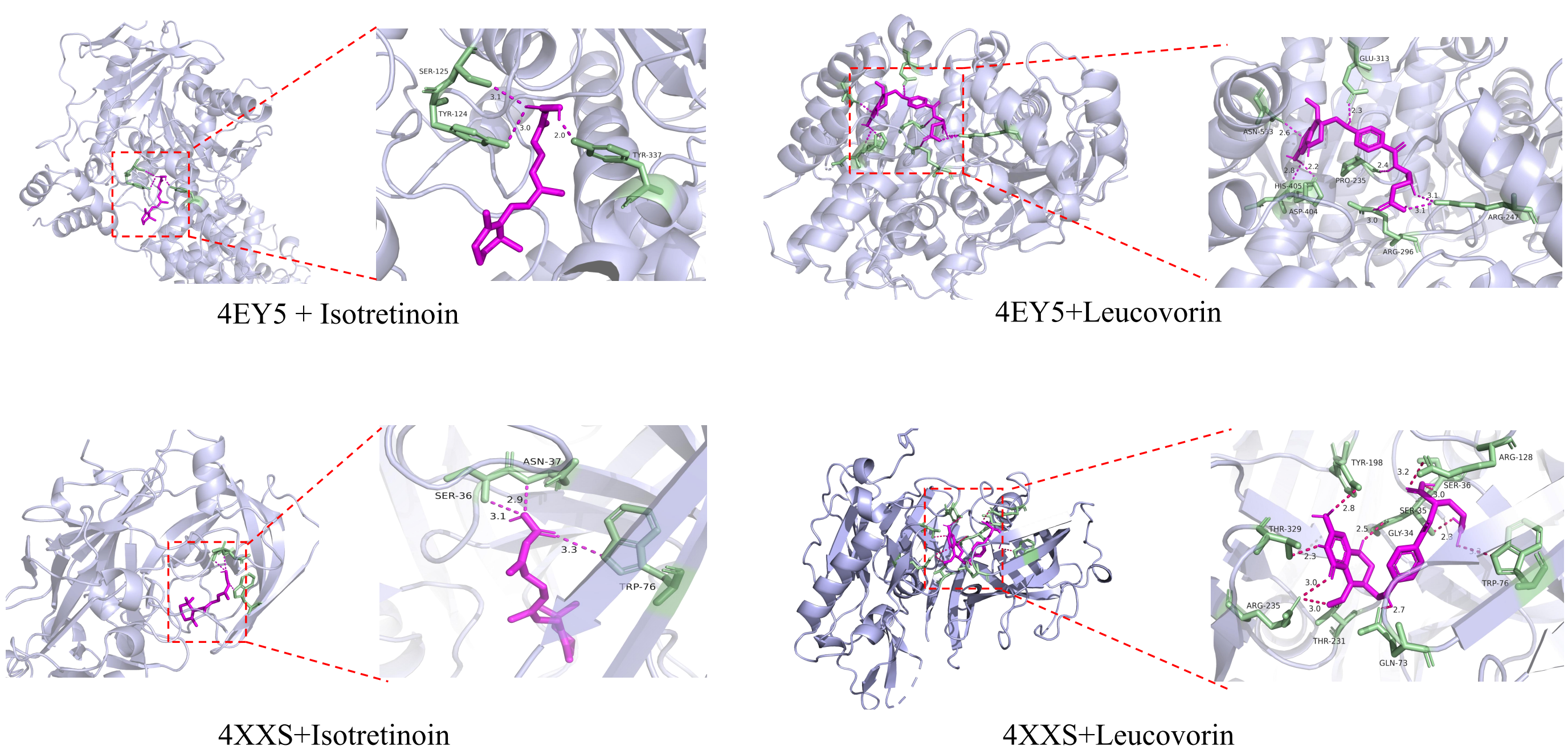

Fig. S7. Additional molecular docking experiment results. Top: Leucovorin-4EY5. Bottom left: Isotretinoin-4XXS. Bottom right: Leucovorin-4XXS. All combinations exhibit favorable binding configurations with negative binding free energies.

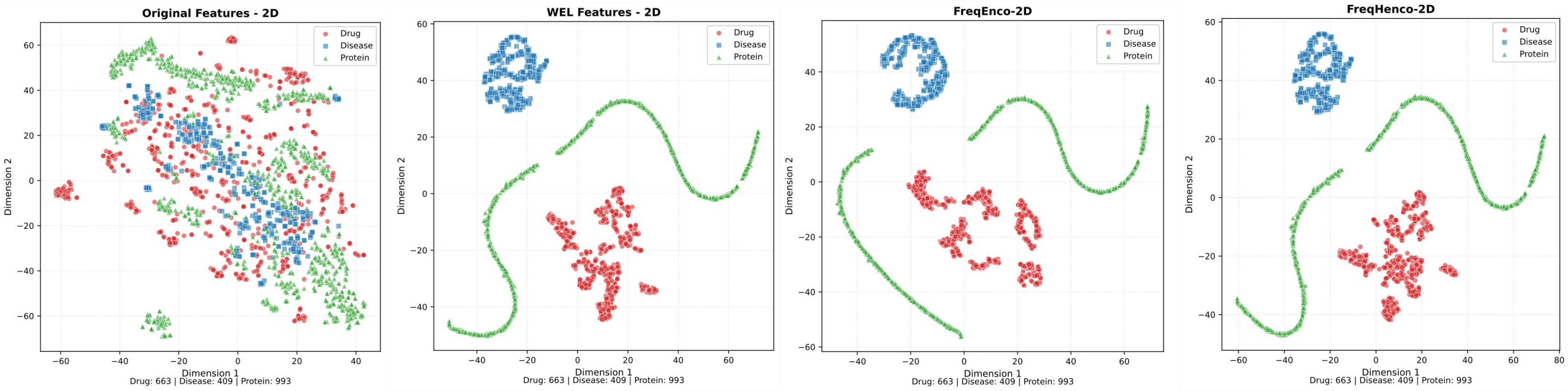

Fig. S8. 2D t-SNE visualization results showing the evolution of node representations across four stages. The progressive clustering and separation demonstrate the effectiveness of each component in the spatial-spectral framework.

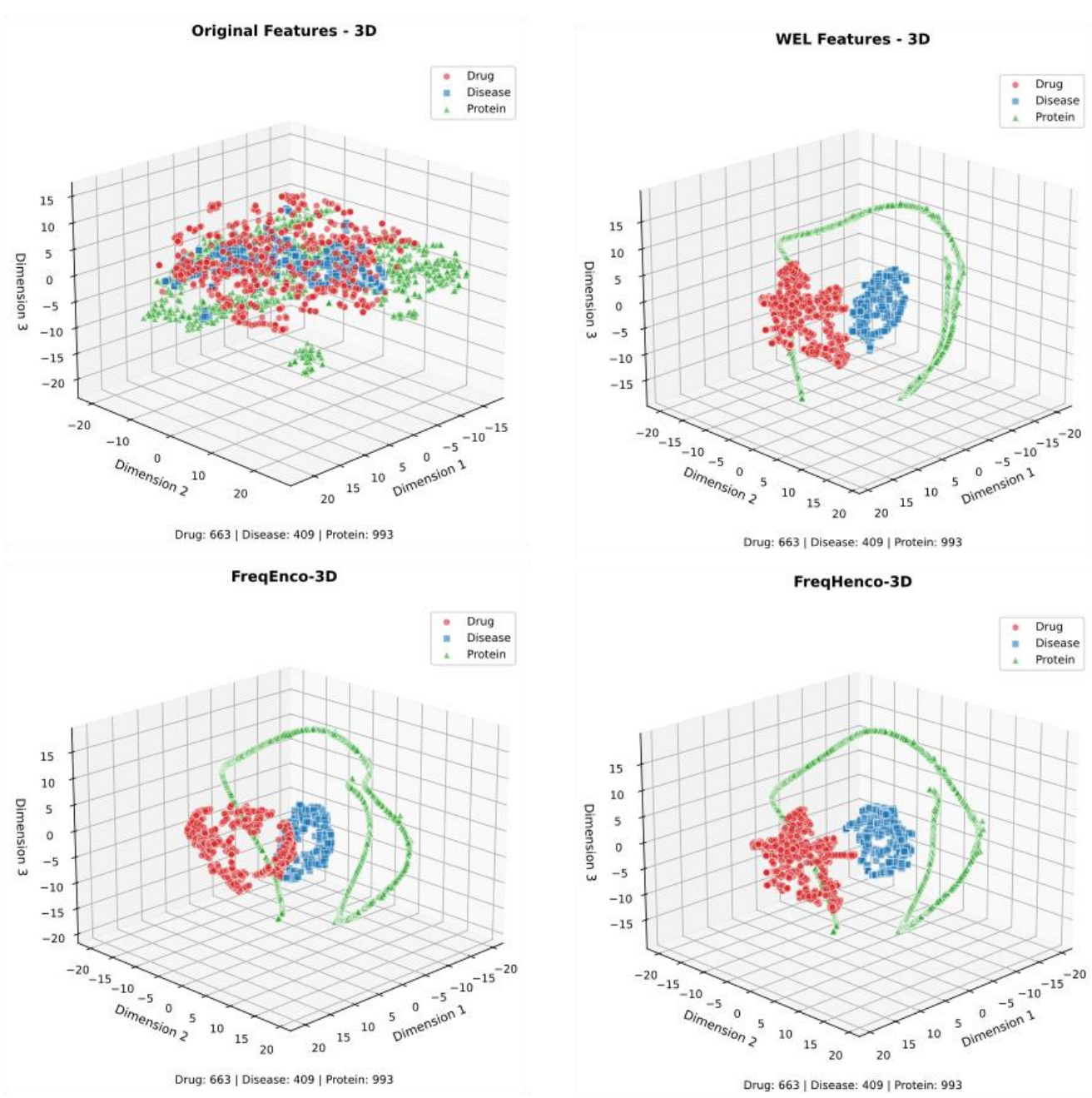

Fig. S9. Visualization and interpretability analysis results.

tegrates network structural information and brings biologically related nodes closer in the feature space.

The FreqEnco stage further refines the homogeneous similarity view. Drug and disease nodes form more compact and better-separated clusters within their corresponding subspaces, showing that spectral encoding on similarity networks enhances global organization and similarity-based discrimination. Finally, FreqHenco jointly encodes drugs, diseases, and proteins on the heterogeneous biological network, leading to the highest level of intra-class compactness and inter-class separability. This indicates that heterogeneous spectral modeling captures long-range biological mechanisms across multiple node types.

Overall, the progressive evolution from mixed representations to organized clusters validates the effectiveness of the spatial-spectral collaborative framework in constructing discriminative biological network representations. Spatial wave evolution captures multi-scale propagation patterns, homogeneous spectral encoding refines similarity-based clustering, and heterogeneous spectral encoding integrates cross-type biological mechanisms.

APPENDIX H
ALGORITHMIC

Algorithm S1 summarizes the overall training procedure of the proposed spatial–spectral framework. The model first projects drug, disease, and protein features into a shared latent space, and then applies spatial wave evolution to capture multi-scale propagation patterns on entity-specific similarity networks. Based on the evolved representations, two complementary spectral views are constructed: FreqEnco encodes global structural information from homogeneous similarity networks, whereas FreqHenco captures cross-type biological semantics from the heterogeneous drug–disease–protein graph. The two views are aligned through contrastive learning and then fused for drug–disease association prediction. The final objective combines the contrastive loss and the classification loss, enabling the model to jointly learn discriminative association representations and cross-view consistency.

APPENDIX I
ALGORITHMIC

Algorithm S1 summarizes the overall training procedure of the proposed spatial–spectral framework. The model first projects drug, disease, and protein features into a shared latent space, and then applies spatial wave evolution to capture multi-scale propagation patterns on entity-specific similarity networks. Based on the evolved representations, two complementary spectral views are constructed: FreqEnco encodes global structural information from homogeneous similarity networks, whereas FreqHenco captures cross-type biological semantics from the heterogeneous drug–disease–protein graph. The two views are aligned through contrastive learning and then fused for drug–disease association prediction. The final objective combines the contrastive loss and the classification loss, enabling the model to jointly learn discriminative association representations and cross-view consistency.

Algorithm S1 Spatial-Spectral Framework for Drug Repositioning

Require: Drug, disease, and protein features X_r, X_d, X_p; similarity networks A_r, A_d, A_p; heterogeneous graph G

Ensure: Trained model for drug–disease association prediction

for epoch $= 1$ to E **do**
  Feature projection
  for entity type $e \in \{r, d, p\}$ **do**
    $H_e^{(0)} = \mathrm{ELU}(X_e W_e^{proj})$
  end for
  Spatial wave evolution
  for entity type $e \in \{r, d, p\}$ **do**
    $\tilde{H}_e = \mathrm{WEL}(H_e^{(0)}, A_e)$
  end for
  Dual-view spectral encoding
  $Z_r^{sim} = \mathrm{FreqEnco}_r(\tilde{H}_r, A_r)$, $Z_d^{sim} = \mathrm{FreqEnco}_d(\tilde{H}_d, A_d)$
  $Z_r^{sem}, Z_d^{sem} = \mathrm{FreqHenco}(\tilde{H}_r, \tilde{H}_d, \tilde{H}_p, G)$
  View alignment and prediction
  $\mathcal{L}^{contrast} = \mathcal{L}^{cross}(Z^{sim}, Z^{sem}) + \mathcal{L}^{intra}(Z^{sim})$
  $Z_r = \mathrm{Linear}_r([Z_r^{sim} \| Z_r^{sem}])$
  $Z_d = \mathrm{Linear}_d([Z_d^{sim} \| Z_d^{sem}])$
  $\hat{y}_{ij} = \mathrm{Predictor}(Z_r(i,:) \odot Z_d(j,:))$
  Compute classification loss $\mathcal{L}^{cls}$
  $\theta \leftarrow \theta - \eta \nabla_\theta [\alpha \mathcal{L}^{contrast} + (1-\alpha)\mathcal{L}^{cls}]$
end for

Appendix J
Robustness under Positive-to-Negative Class Imbalance

In addition to the ablation analysis above, we further evaluate whether the proposed model remains robust when the positive-to-negative sample ratio becomes more imbalanced. Specifically, we conduct 10-fold cross-validation with positive-to-negative ratios of 1:5 and 1:10, in addition to the original 1:1 setting. The complete results are reported in Table S1.

Overall, AUC remains close to 0.99 across different ratios, indicating robust ranking ability. As the proportion of negative samples increases, AUPR decreases because it is sensitive to class prevalence, while threshold-dependent metrics such as precision, recall, and F1 also decline. This trend is expected under more imbalanced test settings and shows that the model remains stable when negative samples become substantially more frequent.

Appendix K
Evaluation Split Protocol and Cold-Start Validation

To clarify the evaluation protocol, the 10-fold cross-validation used in the main experiments was conducted at the drug–disease pair level. Specifically, all known drug–disease associations were used as positive samples, and an equal number of unknown drug–disease pairs were sampled as negative samples, forming a 1:1 positive-to-negative ratio. The resulting positive and negative pairs were then divided into ten stratified folds. Under this setting, the same drug or disease may appear in both training and test folds, but the specific drug–disease pairs in the test fold are never used for training.

To further evaluate the model's ability to generalize to unseen entities, we additionally constructed three cold-start settings. In the Cold Drug setting, all drugs appearing in the test fold are excluded from the training fold. In the Cold Disease setting, all diseases appearing in the test fold are excluded from the training fold. In the Cold Both setting, both drugs and diseases appearing in the test fold are excluded from the training fold. For each cold-start setting, negative samples were generated under the same constraint and kept at the same 1:1 positive-to-negative ratio.

Because Gaussian interaction profile (GIP) similarities are computed from drug–disease association profiles, we further performed an inductive cold-start validation to exclude the possibility that full-matrix GIP features provide additional association information for held-out entities. In this inductive setting, the GIP-based drug and disease features were recomputed separately in each fold using only the training associations. Thus, test drugs or diseases do not receive GIP features derived from their held-out test associations, while all other model settings remain unchanged.

As shown in Table S2, when GIP-based features are recomputed using only training associations, performance decreases as expected, particularly under the Cold Both setting. Nevertheless, the AUC values remain high in most cases, indicating that the proposed model retains meaningful generalization ability under stricter cold-start validation.

Appendix L
Score-Distribution Analysis and Graphon-Augmentation Audit

To further assess whether the high performance reported in Table I reflects genuine discriminative ability rather than structural shortcuts, an overly simple evaluation subset, or implicit label leakage from graphon-based metapath augmentation, we conducted two additional analyses. First, we examined the predicted-score distributions of positive and negative test samples. Second, we performed a code-level audit of the graphon-based metapath augmentation procedure.

A. Score-distribution analysis

We first examined the predicted probability distributions of positive and negative test samples. If the high AUC and recall were caused by unstable threshold behavior or abnormal prediction collapse, the predicted probabilities of positive and negative samples would be expected to overlap heavily. Instead, as summarized in Table S3 and Fig. S10, positive samples consistently receive high predicted probabilities, whereas negative samples are concentrated near low probabilities. The mean separation between positive and negative samples ranges from 0.8315 to 0.8928 across datasets and settings, indicating clear score-level discrimination.

These results suggest that the high ranking performance is associated with a clear separation between positive and

TABLE S1
10-FOLD CROSS-VALIDATION RESULTS UNDER DIFFERENT POSITIVE-TO-NEGATIVE RATIOS (MEAN ± STD).

Dataset	Ratio	AUC	AUPR	Recall	Precision	Accuracy	F1
B-dataset	1:1	0.9953 ± 0.002	0.9940 ± 0.003	0.9972 ± 0.003	0.9374 ± 0.015	0.9652 ± 0.008	0.9663 ± 0.008
	1:5	0.9875 ± 0.0052	0.9310 ± 0.0264	0.9165 ± 0.0745	0.8015 ± 0.0804	0.9452 ± 0.0212	0.8497 ± 0.0474
	1:10	0.9878 ± 0.0037	0.9028 ± 0.0327	0.8877 ± 0.0623	0.7968 ± 0.0454	0.9606 ± 0.0073	0.8374 ± 0.0303
C-dataset	1:1	0.9952 ± 0.003	0.9942 ± 0.004	0.9964 ± 0.003	0.9253 ± 0.020	0.9578 ± 0.011	0.9595 ± 0.011
	1:5	0.9933 ± 0.0027	0.9585 ± 0.0173	0.9877 ± 0.0067	0.8129 ± 0.0436	0.9595 ± 0.0106	0.8912 ± 0.0256
	1:10	0.9937 ± 0.0022	0.9320 ± 0.0213	0.9672 ± 0.0163	0.7568 ± 0.0467	0.9682 ± 0.0084	0.8482 ± 0.0317
F-dataset	1:1	0.9945 ± 0.003	0.9928 ± 0.005	0.9927 ± 0.008	0.9284 ± 0.021	0.9578 ± 0.010	0.9593 ± 0.009
	1:5	0.9922 ± 0.0026	0.9573 ± 0.0154	0.9580 ± 0.0298	0.8194 ± 0.0479	0.9569 ± 0.0105	0.8819 ± 0.0241
	1:10	0.9896 ± 0.0037	0.8961 ± 0.0283	0.9275 ± 0.0704	0.7287 ± 0.0630	0.9608 ± 0.0079	0.8119 ± 0.0336

TABLE S2
INDUCTIVE COLD-START VALIDATION RESULTS AFTER RECOMPUTING GIP-BASED FEATURES USING ONLY TRAINING ASSOCIATIONS IN EACH FOLD. VALUES ARE REPORTED AS MEAN ± STANDARD DEVIATION OVER 10 FOLDS.

Dataset	Setting	AUC	AUPR	Accuracy	Precision	Recall	F1
B-dataset	Cold Drug	0.9870±0.0086	0.9814±0.0140	0.9417±0.0287	0.9108±0.0455	0.9831±0.0225	0.9447±0.0251
B-dataset	Cold Disease	0.9884±0.0054	0.9856±0.0072	0.9419±0.0233	0.9188±0.0376	0.9721±0.0371	0.9437±0.0221
B-dataset	Cold Both	0.9642±0.0400	0.9536±0.0479	0.8672±0.0951	0.8366±0.0927	0.9313±0.1749	0.8678±0.1195
C-dataset	Cold Drug	0.9819±0.0112	0.9770±0.0138	0.9405±0.0291	0.9357±0.0273	0.9472±0.0565	0.9403±0.0311
C-dataset	Cold Disease	0.9723±0.0159	0.9683±0.0173	0.8998±0.0545	0.9286±0.0282	0.8678±0.1190	0.8922±0.0666
C-dataset	Cold Both	0.9756±0.0182	0.9723±0.0217	0.9122±0.0386	0.8944±0.0460	0.9378±0.0642	0.9139±0.0396
F-dataset	Cold Drug	0.9618±0.0409	0.9545±0.0453	0.9011±0.0628	0.9081±0.0654	0.8951±0.0804	0.8999±0.0630
F-dataset	Cold Disease	0.9880±0.0081	0.9858±0.0099	0.9434±0.0233	0.9395±0.0188	0.9486±0.0506	0.9431±0.0248
F-dataset	Cold Both	0.9810±0.0257	0.9778±0.0317	0.9313±0.0652	0.9423±0.0565	0.9218±0.1194	0.9271±0.0770

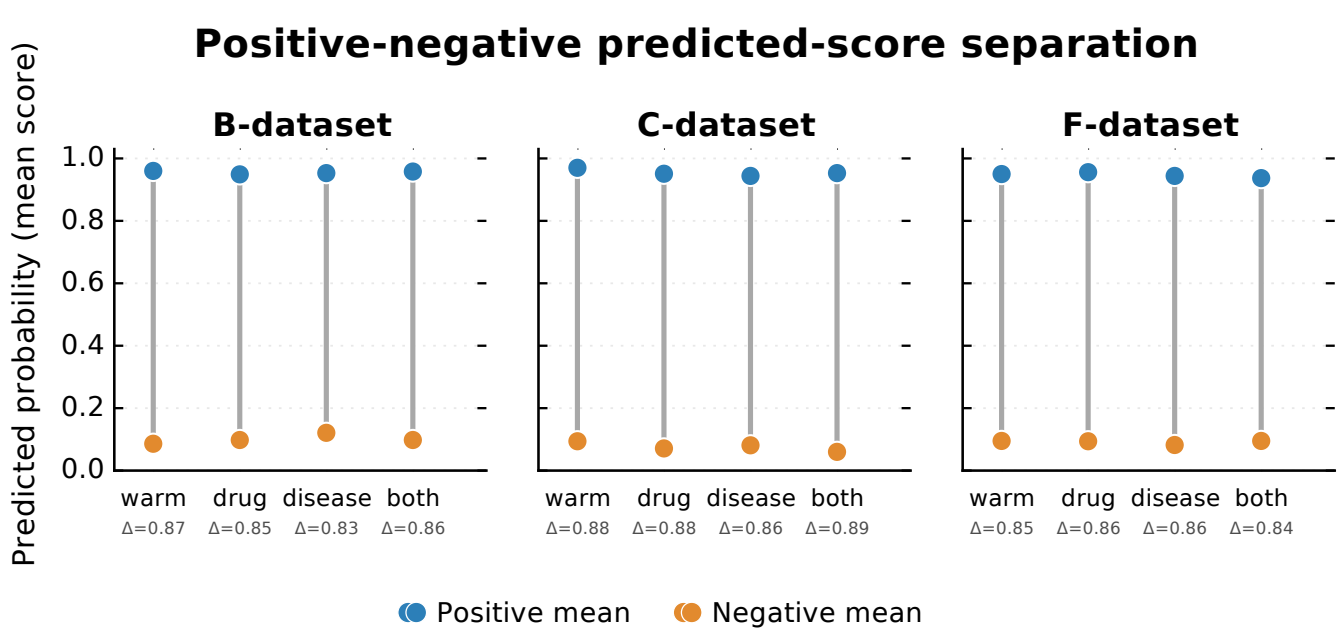

Fig. S10. Predicted-probability separation between positive and negative samples ($\Delta \approx 0.83$–0.89 across protocols, indicating clear class separability).

negative samples under the balanced evaluation setting, rather than being caused by a single decision threshold. The same separation pattern is also observed under the three cold-start settings, further supporting that the model learns discriminative association patterns instead of simply memorizing the original test pairs.

B. Audit of graphon-based metapath augmentation

We further audited the graphon-based metapath augmentation module to examine whether it could introduce implicit test-label leakage. In each fold, the heterogeneous drug–disease–protein graph is constructed using only the training positive drug–disease associations, together with the known drug–protein and disease–protein relationships. The test drug–disease associations are not used when constructing the training graph.

TABLE S3
PREDICTED-SCORE DISTRIBUTION SUMMARY FOR POSITIVE AND NEGATIVE TEST SAMPLES. "ORIGINAL" DENOTES THE ORIGINAL 10-FOLD PAIR-LEVEL EVALUATION SETTING. Δ DENOTES THE MEAN PREDICTED PROBABILITY OF POSITIVE SAMPLES MINUS THAT OF NEGATIVE SAMPLES.

Dataset	Setting	Pos. mean	Neg. mean	Δ
B-dataset	Original	0.9599	0.0857	0.8742
B-dataset	Cold Drug	0.9488	0.0984	0.8504
B-dataset	Cold Disease	0.9526	0.1211	0.8315
B-dataset	Cold Both	0.9578	0.0980	0.8599
C-dataset	Original	0.9698	0.0939	0.8758
C-dataset	Cold Drug	0.9507	0.0706	0.8801
C-dataset	Cold Disease	0.9443	0.0807	0.8636
C-dataset	Cold Both	0.9533	0.0605	0.8928
F-dataset	Original	0.9498	0.0953	0.8545
F-dataset	Cold Drug	0.9556	0.0937	0.8619
F-dataset	Cold Disease	0.9435	0.0824	0.8611
F-dataset	Cold Both	0.9371	0.0954	0.8417

The graphon-based augmentation is then performed on metapath-reachable graphs derived from this training graph. Specifically, graphons are estimated from metapath-induced adjacency matrices such as drug–disease–drug, drug–protein–drug, disease–drug–disease, and disease–protein–disease. The augmented graphs are sampled from the estimated graphons and are used as synthetic metapath-based views. Therefore, the graphon module does not directly access the held-out test drug–disease labels, nor does it insert test drug–disease edges into the augmented training graph.

This design is important because graphon augmentation

operates at the metapath-structure level rather than by copying true held-out associations. The only drug–disease edges available to the graphon estimator in each fold are the training positive associations. Consequently, the graphon augmentation cannot explicitly encode test drug–disease pairs as training edges.

C. Additional validation under stricter settings

The cold-start and inductive cold-start experiments provide further evidence against the possibility that the high performance is mainly driven by memorizing test associations. In the Cold Drug, Cold Disease, and Cold Both settings, the model is evaluated on unseen drugs, unseen diseases, or both. Moreover, in the inductive cold-start setting, GIP-based features are recomputed within each fold using only training associations, so held-out entities do not receive GIP features derived from their test associations.

The performance decreases under the stricter inductive setting, especially in the Cold Both case, which is expected because less association information is available for held-out entities. Nevertheless, the model still maintains strong AUC values in most settings. This indicates that the original high performance is not solely attributable to structural shortcuts or graphon-based leakage, but is also supported by the proposed spatial–spectral mechanism's ability to capture multi-hop similarity propagation and long-range biological association patterns.

References

[1] Y. Luo *et al.*, "A network integration approach for drug-target interaction prediction and computational drug repositioning from heterogeneous information," *Nature Commun.*, vol. 8, no. 1, p. 573, 2017.

[2] X. Zeng, S. Zhu, X. Liu, Y. Zhou, R. Nussinov, and F. Cheng, "DeepDR: A network-based deep learning approach to in silico drug repositioning," *Bioinformatics*, vol. 35, no. 24, pp. 5191–5198, 2019.

[3] Z. Yu, F. Huang, X. Zhao, W. Xiao, and W. Zhang, "Predicting drug–disease associations through layer attention graph convolutional network," *Brief. Bioinf.*, vol. 22, no. 4, p. bbaa243, 2021.

[4] S. Huang, M. Wang, X. Zheng, J. Chen, and C. Tang, "Hierarchical and dynamic graph attention network for drug-disease association prediction," *IEEE J. Biomed. Health Inform.*, vol. 28, no. 4, pp. 2416–2427, 2024.

[5] L. Cai *et al.*, "Drug repositioning based on the heterogeneous information fusion graph convolutional network," *Brief. Bioinf.*, vol. 22, no. 6, p. bbab319, Nov. 2021.

[6] Y. Meng, C. Lu, M. Jin, J. Xu, X. Zeng, and J. Yang, "A weighted bilinear neural collaborative filtering approach for drug repositioning," *Brief. Bioinf.*, vol. 23, no. 2, p. bbab581, 2022.

[7] J. Liu, S. Guan, Q. Zou, H. Wu, P. Tiwari, and Y. Ding, "AMDGT: Attention aware multi-modal fusion using a dual graph transformer for drug–disease associations prediction," *Knowl.-Based Syst.*, vol. 284, p. 111329, 2024.

[8] Z. Wu, S. Pan, F. Chen, G. Long, C. Zhang, and P. S. Yu, "A comprehensive survey on graph neural networks," *IEEE Trans. Neural Netw. Learn. Syst.*, vol. 32, no. 1, pp. 4–24, Jan. 2021.

[9] B.-W. Zhao, L. Hu, Z.-H. You, L. Wang, and X.-R. Su, "HINGRL: Predicting drug–disease associations with graph representation learning on heterogeneous information networks," *Brief. Bioinf.*, vol. 23, no. 1, p. bbab515, 2022.

[10] M.-L. Zhang, B.-W. Zhao, X.-R. Su, Y.-Z. He, Y. Yang, and L. Hu, "RLFDDA: A meta-path based graph representation learning model for drug–disease association prediction," *BMC Bioinf.*, vol. 23, no. 1, p. 516, 2022.

[11] B.-W. Zhao *et al.*, "Drug-disease association prediction using semantic graph and function similarity representation learning over heterogeneous information networks," *Methods*, vol. 220, pp. 106–114, 2023.

[12] B.-W. Zhao, X.-R. Su, P.-W. Hu, Y.-P. Ma, X. Zhou, and L. Hu, "A geometric deep learning framework for drug repositioning over heterogeneous information networks," *Brief. Bioinf.*, vol. 23, no. 6, p. bbac384, 2022.

[13] Y. Zhang, C. Yuan, L. Wang, Y. Chen, Y. Xing, and Y. Sun, "The structure-preserving spectral graph neural network for dual kinase inhibitors and synergy scoring in gastric cancer," *npj Digit. Med.*, vol. 9, art.on.1, 2026.

[14] S. Jin, Y. Zhang, H. Yu, and M. Lu, "SADR: Self-supervised graph learning with adaptive denoising for drug repositioning," *IEEE/ACM Trans. Comput. Biol. Bioinf.*, vol. 21, no. 2, pp. 265–277, 2024.

[15] H. Yu, M. Lu, Z. Li, and Y. Zhang, "Semantic-enhanced graph contrastive learning with adaptive denoising for drug repositioning," *IEEE J. Biomed. Health Inform.*, vol. 29, no. 3, pp. 1635–1643, Mar. 2025.

[16] X. Jia, X. Sun, K. Wang, and M. Li, "DRGCL: Drug repositioning via semantic-enriched graph contrastive learning," *IEEE J. Biomed. Health Inform.*, vol. 29, no. 3, pp. 1656–1667, Mar. 2025.

[17] H. Cui, H. Bi, M. Duan, S. Wang, Y. Qu, and Y. Zhang, "Multi-view contrastive learning for drug repositioning on heterogeneous biological networks," *IEEE J. Biomed. Health Inform.*, vol. 29, no. 9, pp. 6902–6914, 2025.

[18] J. Liu, F. Hu, Q. Zou, P. Tiwari, H. Wu, and Y. Ding, "Drug repositioning by multi-aspect heterogeneous graph contrastive learning and positive-fusion negative sampling strategy," *Inf. Fusion*, vol. 112, p. 102563, 2024.

[19] Y. Zhou *et al.*, "Multi-aspect heterogeneous graph augmentation," in *Proc. ACM Web Conf. (WWW)*, Austin, TX, USA, 2023, pp. 39–48.

[20] W. Zhang *et al.*, "Predicting drug-disease associations by using similarity constrained matrix factorization," *BMC Bioinf.*, vol. 19, no. 1, p. 233, 2018.

[21] H. Luo *et al.*, "Drug repositioning based on comprehensive similarity measures and bi-random walk algorithm," *Bioinformatics*, vol. 32, no. 17, pp. 2664–2671, 2016.

[22] A. Gottlieb, G. Y. Stein, E. Ruppin, and R. Sharan, "PREDICT: A method for inferring novel drug indications with application to personalized medicine," *Mol. Syst. Biol.*, vol. 7, no. 1, p. 496, 2011.
